# Association of adiposity with hemoglobin levels in patients with chronic kidney disease not on dialysis

**DOI:** 10.1007/s10157-017-1501-y

**Published:** 2017-11-04

**Authors:** Hirokazu Honda, Kota Ono, Tadao Akizawa, Kosaku Nitta, Akira Hishida

**Affiliations:** 1 Division of Nephrology, Department of Medicine, Showa University Koto Toyosu Hospital, 5-1-38, Toyosu, Koto-ku, Tokyo, 135-8577 Japan; 2 Clinical Research and Medical Innovation Center, Hokkaido University Hospital, Sapporo, Japan; 3 Division of Nephrology, Department of Medicine, Showa University School of Medicine, Tokyo, Japan; 4 Fourth Department of Internal Medicine, Tokyo Women’s Medical University, Tokyo, Japan; 5 Yaizu City Hospital, Yaizu, Japan

**Keywords:** Abdominal circumference, Body mass index, Erythropoiesis, Inflammation, Nutrition

## Abstract

**Background:**

In the general population, adiposity influences erythropoiesis and iron metabolism. We aimed to assess the relationships between adiposity [estimated by body mass index (BMI) and abdominal circumference (AC)] and biomarkers of erythropoiesis in patients with chronic kidney disease (CKD) not on dialysis.

**Methods:**

A total of 2322 patients from the Chronic Kidney Disease Japan Cohort study were included. Patients were grouped according to BMI (low: < 18.5 kg/m^2^, normal: 18.5–24.5 kg/m^2^, and high: ≥ 25 kg/m^2^) and AC categories (large: ≥ 90 cm for men and ≥ 80 cm for women; small: < 90 cm and < 80 cm, respectively). Body composition and laboratory data were assessed at baseline, and at 1 and 2 years of follow-up.

**Results:**

Multivariate regression analysis of the 3 time-points showed that high BMI and large AC in male patients were significantly associated with higher hemoglobin levels. Hemoglobin levels were lower in female patients with low BMI and small AC than that in female patients with normal BMI and large AC, respectively; however, hemoglobin levels plateaued above a threshold of 25 kg/m^2^ for BMI and 80 cm for AC. While BMI and AC were positively associated with C-reactive protein levels, they were not associated with levels of transferrin saturation, ferritin, and erythropoietin in multivariate models.

**Conclusions:**

Body composition appears to be associated with erythropoiesis; however, adiposity may be only associated with increased erythropoiesis in male patients. In addition, body composition does not appear to hamper iron metabolism in CKD patients not on dialysis.

**Electronic supplementary material:**

The online version of this article (10.1007/s10157-017-1501-y) contains supplementary material, which is available to authorized users.

## Introduction

Anemia is a major complication in patients with advanced chronic kidney disease (CKD) and an independent risk for cardiovascular mortality and end-stage kidney disease [1–3]. While inadequate erythropoietin (EPO) production, or EPO deficiency, in a uremic condition is a main cause of anemia in CKD, a multitude of factors such as malnutrition, chronic inflammation, and a low absorption of nutrients (including iron, folic acid, and vitamin B12) influence erythropoiesis in these patients [4].

Erythropoiesis is more common in overweight and obese individuals than in individuals with normal body weight [5–7]. This phenomenon is thought to be caused by abdominal fat. Abdominal fat is a source of adipokines, such as leptin and adiponectin, and is a cause of diminished insulin response; these factors can lead to erythropoiesis [8–14]. Adiposity is also associated with iron metabolism [15–18]. As abdominal fat is a source of inflammatory cytokines, adiposity causes a state of chronic, low-grade inflammation, which results in the over-production of hepcidin-25 [7]. Iron metabolism is regulated by hepcidin-25; a high level of hepcidin-25 can lead to decreased serum iron levels, increased reticuloendothelial iron accumulation, and/or decreased intestinal iron absorption [19, 20]. Consequently, overweight and obese individuals may develop abnormal iron metabolism. However, the prevalence of anemia in overweight and obese individuals is not higher than that in individuals with normal body weight. Although individuals who are overweight or obese show high levels of iron deficiency, their red blood cell (RBC) count and hemoglobin levels are not always in the anemic range [15, 17].

Thus, adiposity is possibly associated with erythropoiesis and iron deficiency in patients with CKD. However, insulin and adipokine metabolism in CKD patients do not resemble that in the non-CKD population [21–23]. Moreover, patients with advanced CKD often show inadequate EPO production and absolute or functional iron deficiency. Levels of hepcidin-25 increase with increasing CKD severity [24, 25], and patients with advanced CKD may show chronic anemia due to the over-production of hepcidin-25 caused by chronic inflammation [24].

Based on these findings and issues, we hypothesized that adiposity would be associated with erythropoiesis and/or impaired iron metabolism in patients with CKD, and that these associations would be influenced by CKD severity. Therefore, we evaluated the associations between adiposity (estimated by body mass index [BMI] and abdominal circumference [AC]) and biomarkers of erythropoiesis in patients with CKD who were not on dialysis.

## Methods

### Study design

This study was performed as part of the CKD-JAC study, which has been previously described [26, 27]. Briefly, the CKD-JAC study was designed as a multicenter prospective cohort study; 3087 participants who were Japanese patients with CKD not on dialysis were included in the original study [28, 29]. The inclusion criteria were age between 20 and 75 years and low estimated glomerular filtration rate (eGFR) (10–59 mL/min/1.73 m^2^), which was calculated using the following formula: eGFR (mL/min/1.73 m^2^) = 194 age^− 0.287^ serum creatinine^− 1.094^ (× 0.739 for women).

Patients were excluded if they had polycystic kidney disease, human immunodeficiency virus infection, cirrhosis, or active cancer; had undergone cancer treatment within the past 2 years or chronic dialysis; received a transplant; or were pregnant. The protocol was approved by the ethics committee of each participating institution, and the study was conducted in accordance with the Declaration of Helsinki. All patients provided written informed consent. Registration began in September 2007, and 3087 patients were recruited. However, several patients dropped out or were excluded due to exclusion criteria (*n* = 27), unavailable baseline data (*n* = 25), withdrawn consent (*n* = 59), a lack of hospital visits during the observation period (*n* = 4), or death during the study period (*n* = 1). In addition, 5 patients were excluded on the instruction of the attending physician. Therefore, 2966 patients were finally enrolled in the CKD-JAC study [28, 29].

As the present study aimed to assess the impact of adiposity on biomarkers of erythropoiesis and iron metabolism, patients who were treated with an erythropoietin stimulating agent (ESA) were also excluded from analysis. Thus, the BMI cohort comprised 2322 patients with height and weight measurements, and the AC cohort comprised 1625 patients with AC measurements. The WHO international criteria for BMI were used to estimate body composition [30], and the BMI cohort was grouped into 3 categories: low BMI (< 18.5 kg/m^2^), normal BMI (18.5–25 kg/m^2^), and high BMI (≥ 25 kg/m^2^). The AC cohort was grouped into 2 categories using the WHO-recommended cut-off points for the estimation of abdominal obesity in Asian populations [31]: small AC (< 90 cm for men and < 80 cm for women) and large AC (≥ 90 cm for men and ≥ 80 cm for women). AC was measured around the waist, midway between the top of the hip bone and the bottom of the ribs.

### Covariates

Patient characteristics; comorbidities, such as diabetes mellitus (DM) and history of cardiovascular disease; medication use; and routine laboratory data were extracted from the electronic health records at baseline, and at 1 and 2 years of follow-up. Stored frozen serum samples were used to measure intact fibroblast growth factor (intact-FGF23) at the 3 time-points, intact parathyroid hormone and 25-hydroxyvitamin D at baseline, and EPO concentration at 1 year. Intact-FGF23 was measured using a chemiluminescent enzyme immunoassay (Kyowa Medex, Tokyo, Japan), 25-hydroxyvitamin D was measured using a chemiluminescent immunoassay (Liaison 25-hydroxyvitamin D Total assay, DiaSorin, Italy), and EPO concentration was measured using a sandwich radio immunoassay, performed at LSI Medicine Corporation (Tokyo, Japan).

### Statistics

Data are presented as mean ± standard deviation (SD) or medians (range), unless otherwise noted. *p* < 0.05 was considered indicative of statistical significance. Group differences were evaluated using analyses of variance for normally distributed variables, the Kruskal–Wallis test for non-normally distributed variables, and the χ^2^ test for nominal variables. Independent associations between body composition measures and levels of hemoglobin and iron metabolism biomarkers were assessed at baseline, 1 year, and 2 years using multiple linear regression models. In addition, associations body composition measures and levels of hemoglobin, iron metabolism biomarkers, and CRP were assessed using multivariate restricted cubic curves with 4 knots located at the 5th, 35th, 65th and 95th percentiles of BMI or AC. Tests for nonlinearity were performed by comparing a model with a linear term to a model with linear and restricted cubic spline terms. When the test for nonlinearity was not significant, a test for linearity was performed by comparing a model with the linear term to a model without the linear term. Associations between BMI and AC categories and hemoglobin levels were estimated using multivariate repeated-measures analyses. Longitudinal analyses were performed using mixed-effect models with baseline covariates and time-points; the intercept and time-points were also treated as random effects. Interactions between baseline covariates and time-points were considered to reflect a longitudinal effect. All data were analyzed using SAS software (version 9.4, SAS Institute Inc., Cary, North Carolina, USA).

## Results

### Patient characteristics and laboratory findings

A total of 2,322 patients from the Chronic Kidney Disease Japan Cohort (CKD-JAC) study [26, 27] with height and weight measurements were selected as the BMI cohort, and 1,625 patients with AC measurements were selected as the AC cohort. Patient characteristics and laboratory data at baseline are shown according to BMI and AC in Tables 1 and S1, respectively. Patients with high BMI showed increased RBC count and increased levels of hemoglobin, ferritin, and C-reactive protein (CRP) (Tables 1 and 2). In both sexes, the characteristics of patients with a large AC resembled those of patients with a high BMI (Supplementary Tables S1-1 and S1-2). Ferritin levels in patients with a high BMI were higher than those in other BMI categories (Tables 1 and 2). Ferritin levels in female patients, but not male patients, with a large AC were higher than those in patients with a small AC (Supplementary Tables S1-1 and S1-2).

**Table 1:** Male patient characteristics and laboratory findings according to body mass index

	All (*n* = 1497)	Low BMI (*n* = 53)	Normal BMI (*n* = 914)	High BMI (*n* = 530)	p*
Age (years)	60.9 ± 11.2	58.6 ± 14.9	61.8 ± 10.8	59.6 ± 11.3	<0.001
Diabetes mellitus (*n*, %)	603 (40.3)	20 (27.7)	323 (35.3)	260 (49.1)	<0.001
Height (cm)	166.4 ± 6.4	166.2 ± 7.4	166.2 ± 6.3	166.7 ± 6.6	0.396
Weight (kg)	66.7 ± 11.4	48.2 ± 4.6	61.6 ± 6.5	77.4 ± 9.9	<0.001
Body mass index (kg/m^2^)	23.73 (21.67–26.04)	17.45 (16.98–18.07)	22.37 (21.01–23.69)	27.12 (25.82–28.91)	<0.001
Abdominal circumference (cm)	85.5 ± 9.6 [1061]	70.7 ± 4.2	82.6 ± 6.2	95.2 ± 8.3	<0.001
Cause of CKD (*n*, %)					<0.001
CGN	570 (38.1)	21 (39.6)	385 (42.1)	164 (30.9)	
DMN	323 (21.6)	10 (18.9)	162 (17.7)	151 (28.5)	
Nephrosclerosis	350 (23.4)	12 (22.6)	194 (21.2)	144 (27.2)	
Other diseases	254 (17.0)	10 (18.9)	173 (18.9)	71 (13.4)	
CKD stage (*n*, %)					0.282
3A	174 (11.6)	2 (3.8)	102 (11.2)	70 (13.2)	
3B	592 (39.5)	25 (47.2)	350 (38.3)	217 (40.9)	
4	560 (37.4)	19 (35.8)	352 (38.5)	189 (35.7)	
5	171 (11.4)	7 (13.2)	110 (12.0)	54 (10.2)	
History of CVD (yes, %)	394 (26.3)	13 (24.5)	233 (25.5)	148 (27.9)	0.573
ACE inhibitor/ARB (yes, %)	1252 (83.6)	37 (69.8)	750 (82.1)	465 (87.7)	<0.001
Ferrotherapy (*n*, %)	48 (3.2)	2 (3.8)	31 (3.4)	15 (2.8)	<0.001
Red blood cell count (10^4^/μL)	404.8 ± 60.6 [1474]	387.9 ± 63.4	396.9 ± 58.4	420.1 ± 61.1	<0.001
Hemoglobin (g/dL)	12.72 ± 1.84 [1474]	12.16 ± 1.84	12.48 ± 1.76	13.20 ± 1.82	<0.001
Serum albumin (g/dL)	3.95 ± 0.43 [1450]	3.94 ± 0.44	3.97 ± 0.44	3.96 ± 0.42	0.788
Serum creatinine (mg/dL)	2.20 ± 1.01	2.33 ± 1.02	2.20 ± 0.99	2.18 ± 1.03	0.554
eGFR (ml/min/1.73 m^2^)	30.44 ± 11.87	28.57 ± 10.52	30.11 ± 11.69	31.19 ± 12.27	0.129
Serum cystatin C (mg/L)	1.807 ± 0.652 [1397]	1.964 ± 0.603	1.824 ± 0.664	1.764 ± 0.633	0.071
Serum corrected calcium (mg/dL)	9.17 ± 0.43 [1341]	9.14 ± 0.38	9.17 ± 0.42	9.17 ± 0.45	0.843
Serum phosphate (mg/dL)	3.54 ± 0.70 [1313]	3.77 ± 0.81	3.52 ± 0.67	3.52 ± 0.73	<0.001
Intact parathyroid hormone (pg/mL)	74.0 (50.0–114.0) [1397]	101.0 (61.0–140.0)	74.0 (50.0–112.0)	73.0 (50.0–111.0)	0.056^a^
25-Hydroxyvitamin D (ng/mL)	16.20 (10.00–24.05) [1364]	14.35 (9.30–20.20)	17.10 (10.30–25.10)	15.10 (9.70–22.60)	0.012^a^
Fibroblast growth factor 23 (pg/mL)	56.6 (40.5–86.3) [1375]	54.3 (43.2–98.8)	56.6 (39.9–83.5)	56.7 (41.4–91.5)	0.505^a^
Serum iron (μg/dL)	87.7 ± 30.8 [795]	86.1 ± 27.5	87.1 ± 31.2	88.8 ± 30.4	0.745
Total iron-binding capacity (μg/dL)	291.9 ± 49.9 [526]	295.3 ± 91.8	287.5 ± 48.6	299.6 ± 46.4	0.030
Transferrin saturation (%)	31.23 ± 11.73 [525]	29.98 ± 11.56	31.60 ± 12.25	30.68 ± 10.78	0.634
Serum ferritin (ng/mL)	116.50 (64.00–194.45) [756]	93.00 (40.00–148.20)	113.00 (61.00–187.00)	125.50 (74.00–208.00)	0.021^a^
C-reactive protein (mg/dL)	0.100 (0.040–0.200) [1139]	0.080 (0.030–0.200)	0.090 (0.040–0.200)	0.120 (0.050–0.230)	<0.001^a^
Urine albumin-to-creatinine ratio (mg/g Cr)	508.80 (108.20–1367.60) [1369]	671.15 (163.90–1348.20)	433.55 (89.10–1184.20)	619.30 (139.00–1661.60)	0.001

Values are expressed as *n* (%), mean ± SD, or median (interquartile range). *p value for BMI-group differences. The number of participants with non-missing data is shown in []; proportions are based on non-missing data

*BMI* body mass index, *low BMI* < 18.5 kg/m^2^, *normal BMI* 18.5-24.5 kg/m^2^, *high BMI* ≥ 25 kg/m^2^, *CKD* chronic kidney disease, *CGN* chronic glomerulonephritis, *DMN* diabetic nephropathy, *CVD* cardiovascular disease, *ACE inhibitor* angiotensin-converting enzyme inhibitor, *ARB* angiotensin II receptor blocker, *eGFR* estimated glomerular filtration rate

^a^*p* values were calculated using the Kruskal–Wallis test

**Table 2:** Female patient characteristics and laboratory findings according to body mass index

	All (*n* = 825)	Low BMI (*n* = 96)	Normal BMI (*n* = 502)	High BMI (*n* = 227)	p*
Age (years)	58.2 ± 12.2	54.4 ± 14.2	58.5 ± 11.9	59.3 ± 11.7	0.004
Diabetes mellitus (*n*, %)	238 (28.8)	22 (22.9)	112 (22.3)	104 (45.8)	<0.001
Height (cm)	153.8 ± 6.0	155.2 ± 6.4	153.9 ± 6.0	153.1 ± 5.7	0.019
Weight (kg)	54.3 ± 10.4	40.6 ± 5.3	51.5 ± 5.6	66.5 ± 8.2	<0.001
Body mass index (kg/m^2^)	22.54 (19.98–25.44)	17.43 (16.12–18.01)	21.77 (20.13–23.23)	27.69 (26.22–29.61)	<0.001
Abdominal circumference (cm)	80.1 ± 11.8 [553]	66.5 ± 6.5	77.3 ± 8.4	92.4 ± 9.0	<0.001
Cause of CKD (*n*, %)					<0.001
CGN	470 (57.0)	52 (54.2)	307 (61.2)	111 (48.9)	
DMN	109 (13.2)	6 (6.3)	48 (9.6)	55 (24.2)	
Nephrosclerosis	94 (11.4)	9 (9.4)	52 (10.4)	33 (14.5)	
Other diseases	152 (18.4)	29 (30.2)	95 (18.9)	28 (12.3)	
CKD stage (*n*, %)					0.003
3A	104 (12.6)	5 (5.2)	58 (11.6)	41 (18.1)	
3B	306 (37.1)	28 (29.2)	198 (39.4)	80 (35.2)	
4	322 (39.0)	46 (47.9)	197 (39.2)	79 (34.8)	
5	93 (11.3)	17 (17.7)	49 (9.8)	27 (11.9)	
History of CVD (yes, %)	644 (78.1)	8 (8.3)	70 (13.9)	35 (15.4)	0.231
ACE inhibitor/ARB (yes, %)	200 (24.2)	53 (59.4)	386 (76.9)	201 (88.5)	<0.001
Ferrotherapy (*n*, %)	83 (10.1)	16 (16.7)	47 (9.4)	20 (8.8)	0.071
Red blood cell count (10^4^/μL)	382.9 ± 53.9 [809]	360.3 ± 49.3	382.8 ± 54.7	392.7 ± 51.1	<0.001
Hemoglobin (g/dL)	11.64 ± 1.45 [809]	11.08 ± 1.33	11.62 ± 1.43	11.94 ± 1.50	<0.001
Serum albumin (g/dL)	4.00 ± 0.39 [800]	4.02 ± 0.37	4.02 ± 0.38	3.93 ± 0.39	0.011
Serum creatinine (mg/dL)	1.69 ± 0.77	1.96 ± 0.86	1.65 ± 0.73	1.66 ± 0.81	0.001
eGFR (ml/min/1.73 m^2^)	30.53 ± 12.20	26.51 ± 10.95	30.86 ± 12.00	31.50 ± 12.85	0.002
Serum cystatin C (mg/L)	1.712 ± 0.620 [772]	1.926 ± 0.628	1.673 ± 0.612	1.708 ± 0.618	0.002
Serum corrected calcium (mg/dL)	9.28 ± 0.455 [725]	9.22 ± 0.53	9.28 ± 0.43	9.31 ± 0.46	0.348
Serum phosphate (mg/dL)	3.73 ± 0.60 [715]	3.83 ± 0.71	3.71 ± 0.59	3.73 ± 0.57	0.258
Intact parathyroid hormone (pg/mL)	77.0 (54.0–122.0) [773]	102.0 (59.0–160.0)	76.0 (53.0–111.0)	77.0 (56.0–125.5)	0.024^a^
25-Hydroxyvitamin D (ng/mL)	13.45 (8.80–19.00) [754]	12.85 (8.05–18.75)	13.70 (9.40–19.40)	12.90 (8.20–17.90)	<0.012^a^
Fibroblast growth factor 23 (pg/mL)	49.7 (36.6–73.8) [762]	49.6 (36.4–69.8)	48.4 (36.2–73.4)	52.9 (38.4–79.5)	0.266^a^
Serum iron (μg/dL)	79.1 ± 32.5 [446]	79.8 ± 30.4	78.4 ± 30.9	80.2 ± 36.8	0.867
Total iron-binding capacity (μg/dL)	301.7 ± 54.4 [286]	305.4 ± 69.4	300.6 ± 54.5	302.6 ± 45.8	0.877
Transferrin saturation (%)	28.00 ± 12.25 [285]	28.77 ± 13.60	27.35 ± 11.58	29.14 ± 13.12	0.532
Serum ferritin (ng/mL)	60.65 (32.85–120.30) [412]	43.50 (18.45–117.55)	57.64 (31.50–115.50)	76.45 (41.90–126.20)	0.016^a^
C-reactive protein (mg/dL)	0.070 (0.030–0.200) [611]	0.040 (0.020–0.115)	0.060 (0.030–0.150)	0.110 (0.050–0.280)	<0.001^a^
Urine albumin-to-creatinine ratio (mg/g Cr)	430.20 (111.10–1087.50) [761]	406.40 (147.25–817.80)	383.45 (95.20–1032.60)	585.20 (147.10–1495.10)	0.008

Values are expressed as n (%), mean ± SD, or median (inter quartile range). *P value for BMI-group differences. The number of participants with non-missing data is shown in []; proportions are based on non-missing data. ^a^P values were calculated using the Kruskal–Wallis test. BMI: body mass index, low BMI: < 18.5 kg/m^2^, normal BMI: 18.5–24.5 kg/m^2^, high BMI: ≥ 25 kg/m^2^, CKD: chronic kidney disease, CGN: chronic glomerulonephritis, DMN: diabetic nephropathy, CVD: cardiovascular disease, ACE inhibitor: angiotensin-converting enzyme inhibitor, ARB: angiotensin II receptor blocker, eGFR: estimated glomerular filtration rate

### Associations between body composition measures and hemoglobin level

**Fig. 1:**
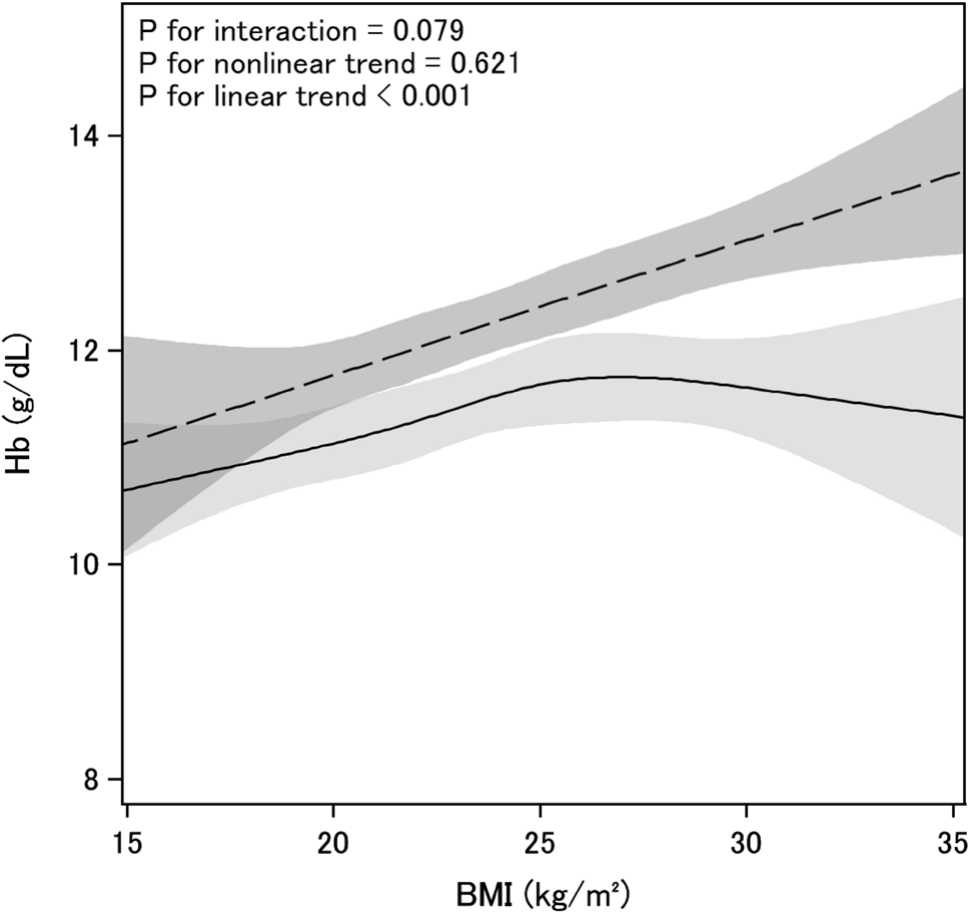
Spline curves show the associations between body mass index (BMI) and hemoglobin (Hb) level at baseline, according to sex [solid lines, female patients (*n* = 219); dashed lines, male patients (*n* = 435)]. Spline curves are adjusted for age (in 10-year increments); diabetes mellitus status; chronic kidney disease stage (3, 4, and 5); levels of albumin, log C-reactive protein, log fibroblast growth factor 23, log ferritin, transferrin saturation, albumin-adjusted calcium, and phosphate; medication use (angiotensin-converting enzyme inhibitor inhibitors and angiotensin II receptor blockers); and ferrotherapy use

Independent associations between body composition and hemoglobin level were evaluated at baseline, and at 1 and 2 years of follow-up in multiple linear regression models, adjusting for various potential cofounders. In addition, multivariate restricted cubic curves were constructed. Hemoglobin levels were significantly increased in male patients with high BMI compared to those in male patients with normal BMI (Table 3), with hemoglobin level increasing linearly with increasing BMI (Fig. 1). Hemoglobin levels were reduced in female patients with low BMI compared to those in female patients with normal BMI (Table 3). Although the association between BMI and hemoglobin level was linear at lower BMI levels in female patients, the spline curve tended to plateau once a BMI threshold of 25 kg/m^2^ was reached (Fig. 1).

**Table 3:** Association between body mass index and hemoglobin levels according to sex, as assessed in multivariate regression models

Baseline	Coefficient (95% confidential interval), p value					
	Male patients			Female patients		
	Model 1 (*n* = 1474)	Model 2 (*n* = 432)	Model 3 (*n* = 431)	Model 1 (*n* = 809)	Model 2 (*n* = 219)	Model 3 (*n* = 214)
Low BMI	− 0.361 (− 0.779, 0.077) *p* = 0.106	− 0.991 (− 1.807, − 0.176) *p* **=** 0.017	− 0.986 (− 1.853, − 0.120) *p* = 0.026	− 0.384 (− 0.682, − 0.086) *p* = 0.012	− 0.685 (− 1.243, − 0.127) *p* = 0.016	**−** 0.638 (− 1.210, − 0.066) *p* = 0.029
Normal BMI	Ref.	Ref.	Ref.	Ref.	Ref.	Ref.
High BMI	0.672 (0.501, 0.843) *p* < 0.001	0.577 (0.296, 0.858) *p* < 0.001	0.588 (0.304, 0.871) *p* < 0.001	0.365 (0.147, 0.583) *p* = 0.001	− 0.016 (− 0.484, 0.473) *p* = 0.942	− 0.333 (− 0.464, 0.399) *p* = 0.881
**1** **year**	Model 1 (*n* = 942)	Model 4 (*n* = 284)	–	Model 1 (*n* = 522)	Model 4 (*n* = 132)	–
Low BMI	− 0.663 (− 1.149, − 0.176) *p* **=** 0.008	− 0.786 (− 1.619, 0.046) *p* = 0.064	–	− 0.553 (− 0.911, − 0.194) *p* = 0.003	− 0.844 (− 1.498, − 0.191) *p* = 0.012	**–**
Normal BMI	Ref.	Ref.	–	Ref.	Ref.	–
High BMI	0.582 (0.373, 0.7491) *p* < 0.001	0.702 (0.316, 1.087) *p* < 0.001	–	0.208 (− 0.050, 0.466) *p* = 0.114	0.289 (− 0.172, 0.750) *p* = 0.217	–
**2** **years**	Model 1 (*n* = 752)	Model 2 (*n* = 219)	–	Model 1 (*n* = 433)	Model 2 (*n* = 119)	–
Low BMI	− 0.589 (− 1.167, − 0.012) *p* = 0.045	− 0.553 (− 1.671, 0.566) *p* = 0.331	–	− 0.675 (− 1.067, − 0.012) *p* < 0.001	− 0.813 (− 1.469, − 0.157) *p* = 0.016	–
Normal BMI	Ref.	Ref.	–	Ref.	Ref.	–
High BMI	0.602 (0.369, 0.834) *p* < 0.001	0.683 (0.271, 1.095) *p* = 0.001	**–**	0.201 (− 0.369, 0.834) *p* = 0.194	− 0.057 (− 0.596, 0.482) *p* = 0.834	–

Hemoglobin level was the dependent factor in models 1-4. The associations between BMI category and hemoglobin level according to sex were adjusted for confounders as follows. Model 1: age, diabetes mellitus status, and chronic kidney disease stage (3, 4, and 5). Model 2: albumin level, C-reactive protein level, transferrin saturation, ferritin level, calcium level corrected by the albumin level, phosphate level, fibroblast growth factor 23 level, urine albumin-to-creatinine ratio, angiotensin-converting enzyme inhibitor use, angiotensin II receptor blocker use, erythropoiesis stimulating agent use, ferrotherapy use, diet therapy, and the confounders in model 1. Model 3: 25-hydroxyvitamin D level, intact parathyroid hormone level, and the confounders in model 2. Model 4: albumin level, C-reactive protein level, transferrin saturation, ferritin level, calcium level corrected by the albumin level, phosphate level, fibroblast growth factor 23 level, EPO concentration, angiotensin-converting enzyme inhibitor use, angiotensin II receptor blocker use, erythropoiesis stimulating agent use, ferrotherapy use, diet therapy, and the confounders in model 1

*Low BMI* < 18.5 kg/m^2^, *normal BMI* 18.5–24.5 kg/m^2^, *high BMI* ≥ 25 kg/m^2^

The associations between AC and hemoglobin level resembled those for BMI and hemoglobin level (Supplementary Table S2). Hemoglobin levels were significantly increased in male patients with large AC compared to those in male patients with small AC (Supplementary Table S2). Although hemoglobin levels increased linearly with increasing AC in both sexes (Supplementary Figure S1), hemoglobin levels plateaued once an AC threshold of 80 cm was reached in female patients (Supplementary Figure S1).

Given the reduced number of patients in more complicated models, the patient characteristics and laboratory data were compared between those included and excluded from Model 2. The results are provided in Supplementary Tables S3-1 and S3-2.

Multivariate repeated-measures analyses were performed to evaluate cross-sectional and longitudinal effects in the associations between baseline body composition and hemoglobin levels at 3 time-points (baseline, 1 year of follow-up, and 2 years of follow-up). Male patients with low BMI, male patients with high BMI, and male patients with large AC showed independent associations with baseline hemoglobin level as cross-sectional effects (Supplementary Tables S4 and S5). These associations did not change 1 and 2 years later, as indicated in the analysis of longitudinal effects (Supplementary Tables S4 and S5).

### Associations between body composition measures and hemoglobin levels according to CKD stage

Spline curves for the association between BMI and hemoglobin level were linear in patients with stages 3 and 4 CKD, but not in patients with stage 5 CKD (Supplementary Figure S2). In addition, the spline curves for the association between hemoglobin levels and AC according to CKD stage resembled those for BMI (Supplementary Figure S3).

### Association between body composition and erythropoietin concentration at 1 year of follow-up

EPO concentrations at 1 year of follow-up according to body composition and sex are provided in Supplementary Table S6. In the multivariate regression models, there were no significant associations between EPO levels and either BMI or AC (Supplementary Table S7, models 2 and 4; Supplementary Fig. 4). However, linear associations between EPO level and both BMI and AC were confirmed in patients with stage 3 CKD; these associations disappeared in patients with stages 4 and 5 CKD (Supplementary Figure S5).

### Association between body composition and levels of transferrin saturation, ferritin, and C-reactive protein

In multiple linear regression analyses, AC was not associated with transferrin saturation and ferritin levels at baseline, and at 1 and 2 years of follow-up (Supplementary Tables S8 and S9). In contrast, BMI was associated with ferritin levels (Tables 1 and 2). Spline curves showing the associations between body composition measures and transferrin saturation and ferritin levels, are shown in Supplementary Figures S6 (BMI) and S7 (AC). Log-transformed CRP levels increased linearly with increasing BMI and AC at baseline in patients at all stages of CKD (Supplementary Figure S8).

## Discussion

To our knowledge, the present study is the first to analyze the association between body composition and biomarkers of erythropoiesis in patients with CKD who are not on dialysis. The present results demonstrate that BMI and AC are positively associated with hemoglobin levels in patients with CKD. Hemoglobin levels in male patients significantly increased with increasing BMI and AC, and were accompanied by higher CRP levels. This was not the case in female patients. Although hemoglobin levels increased with increased BMI and AC in female patients with BMI less than 25 kg/m^2^ or small AC, hemoglobin levels plateaued once BMI reached 25 kg/m^2^ or the AC reached 80 cm.

In an analysis of data from the third National Health and Nutrition Examination Survey (NHANES), conducted in the USA (*n* = 14,846), BMI did not affect hemoglobin levels; however, the association not assessed according to sex [17]. Kohno et al. analyzed a Japanese cohort of 1029 patients and found that BMI was positively associated with biomarkers of erythropoiesis in a post hoc analysis by sex [32]. Vuong et al. demonstrated similar findings in 6766 NHANES patients; hemoglobin levels increased with increasing AC in both male and female patients [6]. Traissac et al. reported that, in a non-CKD cohort of 1689 female patients and 930 male patients, the prevalence of anemia (hemoglobin < 12.0 g/dL) was significantly higher in females with BMI ≥ 25 kg/m^2^ than in males with BMI ≥ 25 kg/m^2^ [33]. Thus, in general, BMI and AC appear to be positively associated with hemoglobin levels in the non-CKD population. However, these associations are likely to be attenuated in females with BMI higher than or equal to the threshold of 25 kg/m^2^. Overall, the association between hemoglobin levels and BMI appears to be maintained in patients with CKD, even though the hemoglobin levels are in the anemic range.

The associations between body composition and hemoglobin levels were affected by CKD severity, and disappeared once CKD reached stage 5. This may be due to inadequate EPO production and erythropoiesis in the uremic condition. EPO concentration was not associated with BMI or AC in the multivariate regression models adjusting for CKD stage; however, EPO levels increased with increasing BMI and AC in patients with stage 3 CKD. Thus, adiposity may be associated with increased EPO production in stage 3 CKD, and this effect may be diminished in stage 4 CKD and beyond.

In both sexes, hemoglobin levels were higher in patients with normal BMI than in those with low BMI. This might be linked to undernourishment. However, it is not clear why hemoglobin levels did not increase with increasing adiposity in female patients, as levels of EPO and iron metabolism biomarkers increased with increase in BMI and AC to a similar extent in both sexes. Sex hormones could play a role; testosterone has been reported to have a stimulatory effect on erythropoiesis [34]. Testosterone levels in male patients with high BMI between 25 and 30 kg/m^2^ appear to be similar to those in male patients with normal BMI, while the levels in male patients with BMI > 30 kg/m^2^ were decreased compared to those in male patients with normal BMI [35]. Thus, testosterone might stimulate erythropoiesis in male patients with BMI ≤ 30 kg/m^2^; however, erythropoiesis might be associated with other unknown mechanisms in men with BMI > 30 kg/m^2^. Increased CKD stage may influence testosterone levels; the prevalence of hypogonadism (testosterone < 10 nmol/L) is 17% in stages 1–2 CKD, 34% in stage 3 CKD, 38% in stage 4 CKD, and 57% in stage 5 CKD [36]. Therefore, an effect of testosterone might be sustained during CKD stages 3 and 4.

Adiposity enhances low iron status and increases CRP and ferritin levels in the non-CKD population [15–17]; thus, adiposity-related inflammation can cause functional iron deficiency. However, we were not able to confirm this association in patients with CKD. Although CRP levels increased with increasing BMI and AC in stages 3–5 CKD, and ferritin levels increased according to BMI category, BMI and AC did not affect transferrin saturation levels in patients with CKD. Ausk et al. reported that ferritin levels in individuals in the general population with BMI between 25 and 30 kg/m^2^ were higher than in those with BMI > 25 kg/m^2^ [17]. Similarly, Cepeda-Lopez et al. reported that CRP levels and iron status were associated with BMI, with higher CRP levels in females with BMI between 25 and 30 kg/m^2^ than in females with BMI between 18.5 and 24.5 kg/m^2^. However, iron status in the 25–30 kg/m^2^ BMI group resembled that of the 18.5–24.5 kg/m^2^ BMI group, and iron deficiency rose in patients with a BMI ≥ 30 kg/m^2^ [15]. Thus, iron deficiency may not have been evident in the present study due to the smaller numbers of participants with BMI ≥ 30 kg/m^2^.

The present study has several limitations. The patient cohort was relatively small, and there was missing biomarker data, especially for iron metabolism. Several patients were excluded from the 1- and 2-year follow-up, as patients in advanced stages of CKD at baseline were referred for dialysis and did not visit our hospital after completing the baseline measurements. Thus, the patients who left the study may have affected our findings at later time periods. In addition, anemia was managed according to the Japanese guidelines for anemia in patients with CKD [37], and strategies for anemia management, such as ESA therapy and ferrotherapy, were determined by the patient’s primary doctors. Moreover, there was an uneven distribution of CKD stages in the BMI and AC groups in the present study. Finally, we cannot speak to the pathogenesis of increased erythropoiesis with increased adiposity, as we did not assess causal associations.

In conclusion, body composition appears to influence erythropoiesis; however, adiposity may be only associated with increased erythropoiesis in male patients. In contrast, body composition does not appear to hamper iron metabolism in patients with CKD who are not on dialysis. Further research is required, with a larger cohort, to confirm the impact of adiposity on erythropoiesis.

## Electronic supplementary material

Below is the link to the electronic supplementary material.

Supplementary Figure S1. Spline curves show the associations between abdominal circumference and hemoglobin (Hb) level at baseline, according to sex (solid lines, female patients [n = 193]; dashed lines, male patients [n = 394]). Spline curves are adjusted for age (in 10-year increments); diabetes mellitus status; chronic kidney disease stage (3, 4, and 5); levels of albumin, log C-reactive protein, log fibroblast growth factor 23, log ferritin, transferrin saturation, albumin-adjusted calcium, and phosphate; medication use (angiotensin-converting enzyme inhibitor inhibitors and angiotensin II receptor blockers); and ferrotherapy use (PPTX 4822 kb)

Supplementary Figure S2. Spline curves show the associations between body mass index (BMI) and hemoglobin (Hb) level at baseline according to chronic kidney disease (CKD) stage and sex (solid lines, female patients [stage 3, n = 108; stage 4, n = 82; stage 5, n = 29]; dashed lines, male patients [stage 3, n = 220; stage 4, n = 162; stage 5, n = 53]). Spline curves are adjusted for age (in 10-year increments); diabetes mellitus status; levels of albumin, log C-reactive protein, log fibroblast growth factor 23, log ferritin, transferrin saturation, albumin-adjusted calcium, and phosphate; medication use (angiotensin-converting enzyme inhibitor inhibitors and angiotensin II receptor blockers); and ferrotherapy use (PPTX 14420 kb)

Supplementary Figure S3. Spline curves show the associations between abdominal circumference and hemoglobin (Hb) level at baseline according to chronic kidney disease (CKD) stage and sex (solid lines, female patients [stage 3, n = 98; stage 4, n = 68; stage 5, n = 27]; dashed lines, male patients [stage 3, n = 194; stage 4, n = 150; stage 5, n = 50]). Spline curves are adjusted for age (in 10-year increments); diabetes mellitus status; levels of albumin, log C-reactive protein, log fibroblast growth factor 23, log ferritin, transferrin saturation, albumin-adjusted calcium, and phosphate; medication use (angiotensin-converting enzyme inhibitor inhibitors and angiotensin II receptor blockers); and ferrotherapy use (PPTX 14305 kb)

Supplementary Figure S4. Spline curves show the associations between body mass index (BMI) (A) and abdominal circumference (B) and erythropoietin (EPO) level at 1 year, according to sex (solid lines, female patients [BMI, n = 132; abdominal circumference, n = 124]; dashed lines, male patients [BMI, n = 284; abdominal circumference, n = 270]). Spline curves are adjusted for age (in 10-year increments); diabetes mellitus status; chronic kidney disease stage (3, 4, and 5); levels of albumin, log C-reactive protein, log fibroblast growth factor 23, log ferritin, transferrin saturation, albumin-adjusted calcium, and phosphate; medication use (angiotensin-converting enzyme inhibitor inhibitors and angiotensin II receptor blockers); and ferrotherapy use (PPTX 9607 kb)

Supplementary Figure S5. Spline curves show the associations between body mass index (BMI) (A) and abdominal circumference (B) and erythropoietin (EPO) levels at 1 year, according to the chronic kidney disease stage (CKD) and sex (solid lines, female patients [BMI: stage 3, n = 63; stage 4, n = 53; stage 5, n = 16], [abdominal circumference: stage 3, n = 62; stage 4, n = 46; stage 5, n = 16]; dashed lines, male patients [BMI: stage 3, n = 143; stage 4, n = 108; stage 5, n = 33], [abdominal circumference: stage 3, n = 133; stage 4, n = 104; stage 5, n = 33]). Spline curves are adjusted for age (in 10-year increments); diabetes mellitus status; levels of albumin, log C-reactive protein, log fibroblast growth factor 23, log ferritin, transferrin saturation, albumin-adjusted calcium, and phosphate; medication use (angiotensin-converting enzyme inhibitor inhibitors and angiotensin II receptor blockers); and ferrotherapy use (PPTX 28742 kb)

Supplementary Figure S6. Spline curves show the associations between body mass index (BMI) and transferrin saturation (TSAT) (A) and ferritin levels (B) at baseline, according to sex as well as chronic kidney disease (CKD) stage (solid lines, female patients [TSAT: all, n = 219; stage 3, n = 108; stage 4, n = 82; stage 5, n = 29], [ferritin: all, n = 219; stage 3, n = 108; stage 4, n = 82; stage 5, n = 29]; dashed lines, male patients [TSAT: all, n = 435; stage 3, n = 220; stage 4, n = 162; stage 5, n = 53], [ferritin: all, n = 435; stage 3, n = 220; stage 4, n = 162; stage 5, n = 53]). Spline curves are adjusted for age (in 10-year increments); diabetes mellitus status; CKD stage (3, 4, and 5); levels of albumin, log C-reactive protein, log fibroblast growth factor 23, albumin-adjusted calcium, and phosphate; medication use (angiotensin-converting enzyme inhibitor inhibitors and angiotensin II receptor blockers); and ferrotherapy use. Spline curves are additionally adjusted for log ferritin level in the transferrin saturation analysis, and for transferrin saturation in the ferritin analysis (PPTX 38202 kb)

Supplementary Figure S7. Spline curves show the associations between abdominal circumference and transferrin saturation (TSAT) (A) and ferritin levels (B) at baseline, according to sex as well as chronic kidney disease (CKD) stage (solid lines, female patients [TSAT: all, n = 193; stage 3, n = 98; stage 4, n = 68; stage 5, n = 27], [ferritin: all, n = 193; stage 3, n = 98; stage 4, n = 68; stage 5, n = 27]; dashed lines, male patients [TSAT: all, n = 394; stage 3, n = 194; stage 4, n = 150; stage 5, n = 50], [ferritin: all, n = 394; stage 3, n = 194; stage 4, n = 150; stage 5, n = 50]). Spline curves are adjusted for age (in 10-year increments); diabetes mellitus status; CKD stage (3, 4, and 5); levels of albumin, log C-reactive protein, log fibroblast growth factor 23, albumin-adjusted calcium, and phosphate; medication use (angiotensin-converting enzyme inhibitor inhibitors and angiotensin II receptor blockers); and ferrotherapy use. Spline curves are additionally adjusted for log ferritin level in the transferrin saturation analysis, and for transferrin saturation in the ferritin analysis (PPTX 38051 kb)

Supplementary Figure S8. Spline curves show the associations between body mass index (BMI) (A) and abdominal circumference (B) and high-sensitivity C-reactive protein (CRP) level at baseline according to sex as well as chronic kidney disease (CKD) stage (solid lines, female patients [BMI: all, n = 502; stage 3, n = 252; stage 4, n = 186; stage 5, n = 64], [abdominal circumference: all, n = 408; stage 3, n = 205; stage 4, n = 151; stage 5, n = 52]; dashed lines, male patients [BMI: all, n = 963; stage 3, n = 481; stage 4, n = 362; stage 5, n = 120], [abdominal circumference: all, n = 789; stage 3, n = 391; stage 4, n = 299; stage 5, n = 99]). Spline curves are adjusted for age (in 10-year increments); diabetes mellitus status; CKD stage (3, 4, and 5); levels of albumin, log fibroblast growth factor 23, albumin-adjusted calcium, and phosphate; medication use (angiotensin-converting enzyme inhibitor inhibitors and angiotensin II receptor blockers); and ferrotherapy use (PPTX 38197 kb)

Supplementary material 9 (DOCX 31 kb)

Supplementary material 10 (DOCX 32 kb)

Supplementary material 11 (DOCX 27 kb)

Supplementary material 12 (DOCX 30 kb)

Supplementary material 13 (DOCX 29 kb)

Supplementary material 14 (DOCX 27 kb)

Supplementary material 15 (DOCX 24 kb)

Supplementary material 16 (DOCX 22 kb)

Supplementary material 17 (DOCX 25 kb)

Supplementary material 18 (DOCX 34 kb)

Supplementary material 19 (DOCX 30 kb)
