# Correction to: Association of adiposity with hemoglobin levels in patients with chronic kidney disease not on dialysis

**DOI:** 10.1007/s10157-017-1519-1

**Published:** 2018-02-06

**Authors:** Hirokazu Honda, Kota Ono, Tadao Akizawa, Kosaku Nitta, Akira Hishida

**Affiliations:** 1 Division of Nephrology, Department of Medicine, Showa University Koto Toyosu Hospital, 5-1-38, Toyosu, Koto-ku, Tokyo, 135-8577 Japan; 2 Clinical Research and Medical Innovation Center, Hokkaido University Hospital, Sapporo, Japan; 3 Division of Nephrology, Department of Medicine, Showa University School of Medicine, Tokyo, Japan; 4 Fourth Department of Internal Medicine, Tokyo Women’s Medical University, Tokyo, Japan; 5 Yaizu City Hospital, Yaizu, Japan

## Correction to: Clinical and Experimental Nephrology 10.1007/s10157-017-1501-y

The article Association of adiposity with hemoglobin levels in patients with chronic kidney disease not on dialysis, written by Hirokazu Honda, Kota Ono, Tadao Akizawa, Kosaku Nitta and Akira Hishida, was originally published electronically on the publisher’s internet portal (currently springerlink) on November 4, 2017 without open access. With the author(s)’ decision to opt for Open Choice, the copyright of the article changed on February 6, 2018 to © The Author(s) [2017] and the article is forthwith distributed under the terms of the Creative Commons Attribution 4.0 International License (http://creativecommons.org/licenses/by/4.0/), which permits use, duplication, adaptation, distribution and reproduction in any medium or format, as long as you give appropriate credit to the original author(s) and the source, provide a link to the Creative Commons license and indicate if changes were made. The original article was corrected.

