# Supplementary figures and images for: Association of adiposity with hemoglobin levels in patients with chronic kidney disease not on dialysis
Source: Clin Exp Nephrol. 2017 Nov 4;22(3):638–46. doi: 10.1007/s10157-017-1501-y (PMC5956024; doi:10.1007/s10157-017-1501-y)

## Slide 1
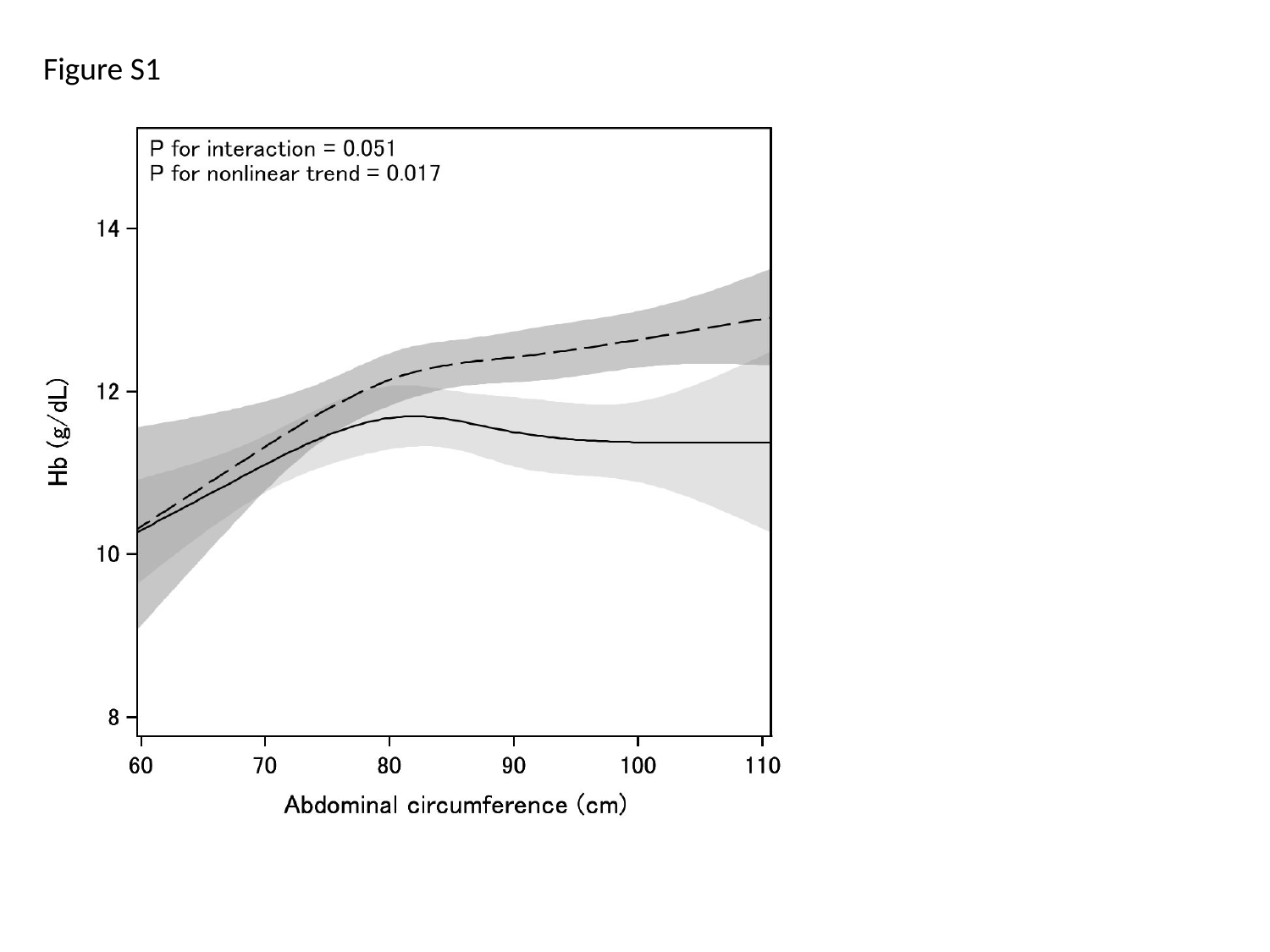

Figure S1

Supplement: Supplementary file 1 — Supplementary Figure S1. Spline curves show the associations between abdominal circumference and hemoglobin (Hb) level at baseline, according to sex (solid lines, female patients [n = 193]; dashed lines, male patients [n = 394]). Spline curves are adjusted for age (in 10-year increments); diabetes mellitus status; chronic kidney disease stage (3, 4, and 5); levels of albumin, log C-reactive protein, log fibroblast growth factor 23, log ferritin, transferrin saturation, albumin-adjusted calcium, and phosphate; medication use (angiotensin-converting enzyme inhibitor inhibitors and angiotensin II receptor blockers); and ferrotherapy use (PPTX 4822 kb) [file 10157_2017_1501_MOESM1_ESM.pptx]

## Slide 1
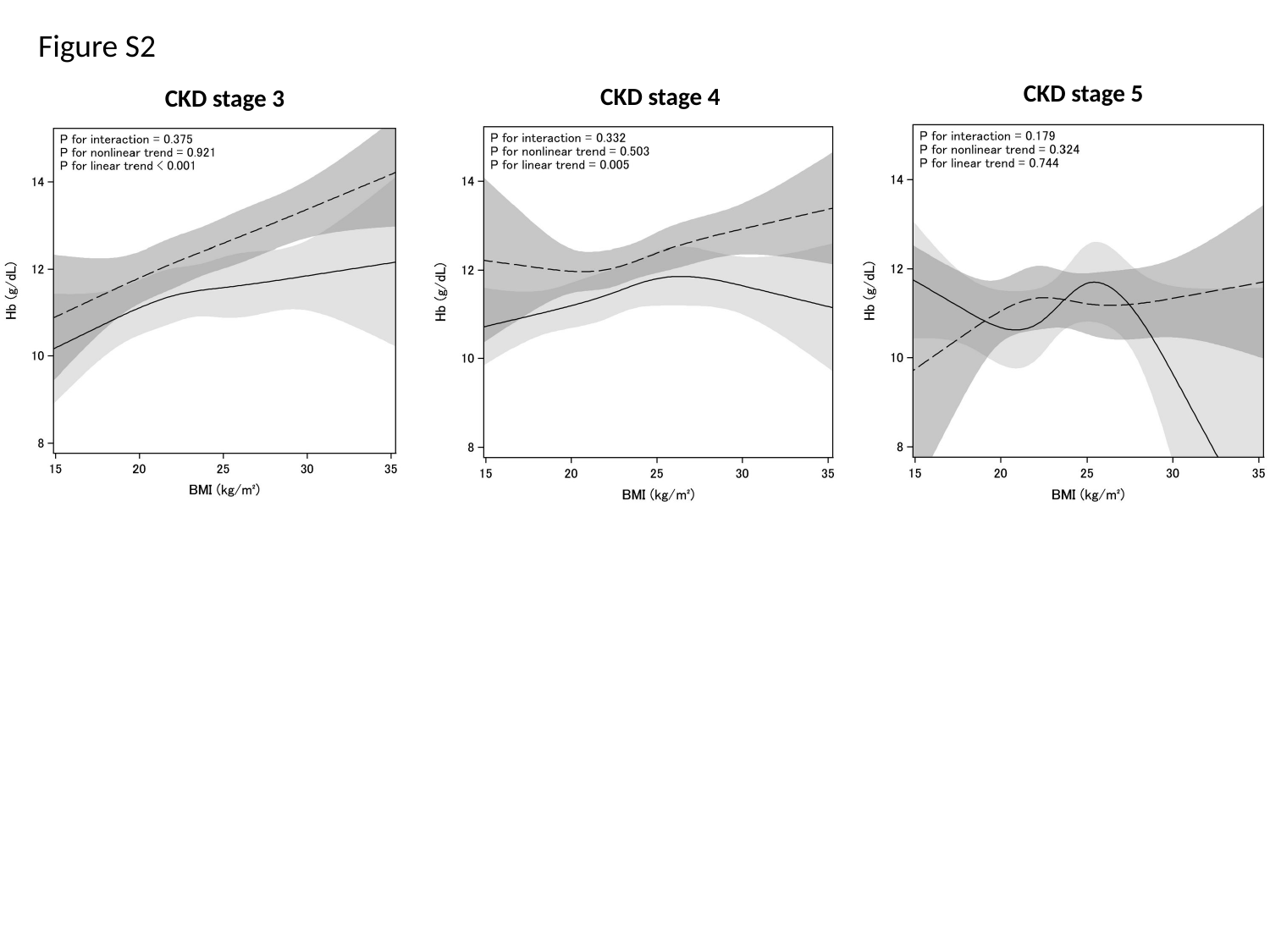

Figure S2
CKD stage 5
CKD stage 4
CKD stage 3

Supplement: Supplementary file 2 — Supplementary Figure S2. Spline curves show the associations between body mass index (BMI) and hemoglobin (Hb) level at baseline according to chronic kidney disease (CKD) stage and sex (solid lines, female patients [stage 3, n = 108; stage 4, n = 82; stage 5, n = 29]; dashed lines, male patients [stage 3, n = 220; stage 4, n = 162; stage 5, n = 53]). Spline curves are adjusted for age (in 10-year increments); diabetes mellitus status; levels of albumin, log C-reactive protein, log fibroblast growth factor 23, log ferritin, transferrin saturation, albumin-adjusted calcium, and phosphate; medication use (angiotensin-converting enzyme inhibitor inhibitors and angiotensin II receptor blockers); and ferrotherapy use (PPTX 14420 kb) [file 10157_2017_1501_MOESM2_ESM.pptx]

## Slide 1
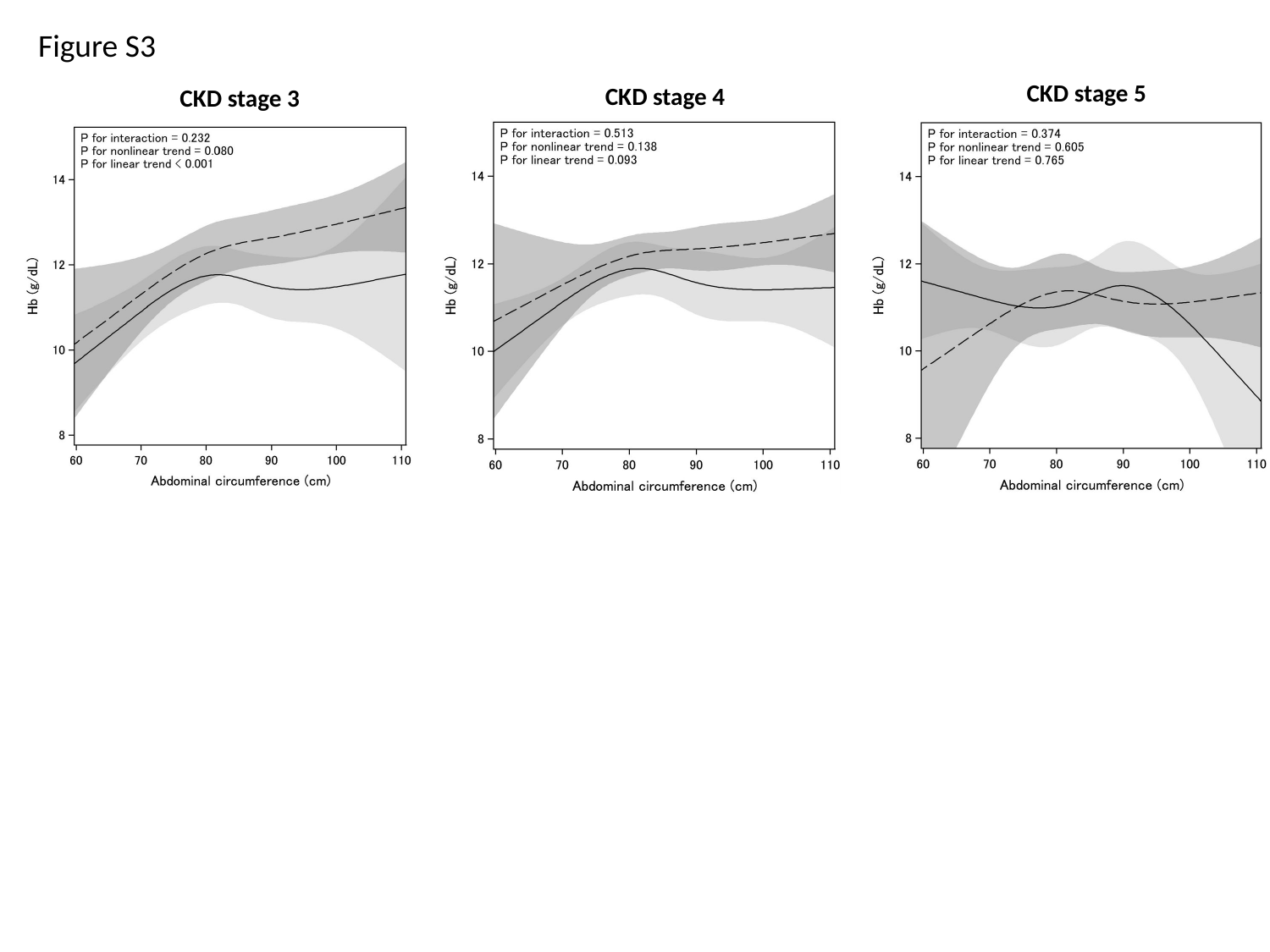

Figure S3
CKD stage 5
CKD stage 4
CKD stage 3

Supplement: Supplementary file 3 — Supplementary Figure S3. Spline curves show the associations between abdominal circumference and hemoglobin (Hb) level at baseline according to chronic kidney disease (CKD) stage and sex (solid lines, female patients [stage 3, n = 98; stage 4, n = 68; stage 5, n = 27]; dashed lines, male patients [stage 3, n = 194; stage 4, n = 150; stage 5, n = 50]). Spline curves are adjusted for age (in 10-year increments); diabetes mellitus status; levels of albumin, log C-reactive protein, log fibroblast growth factor 23, log ferritin, transferrin saturation, albumin-adjusted calcium, and phosphate; medication use (angiotensin-converting enzyme inhibitor inhibitors and angiotensin II receptor blockers); and ferrotherapy use (PPTX 14305 kb) [file 10157_2017_1501_MOESM3_ESM.pptx]

## Slide 1
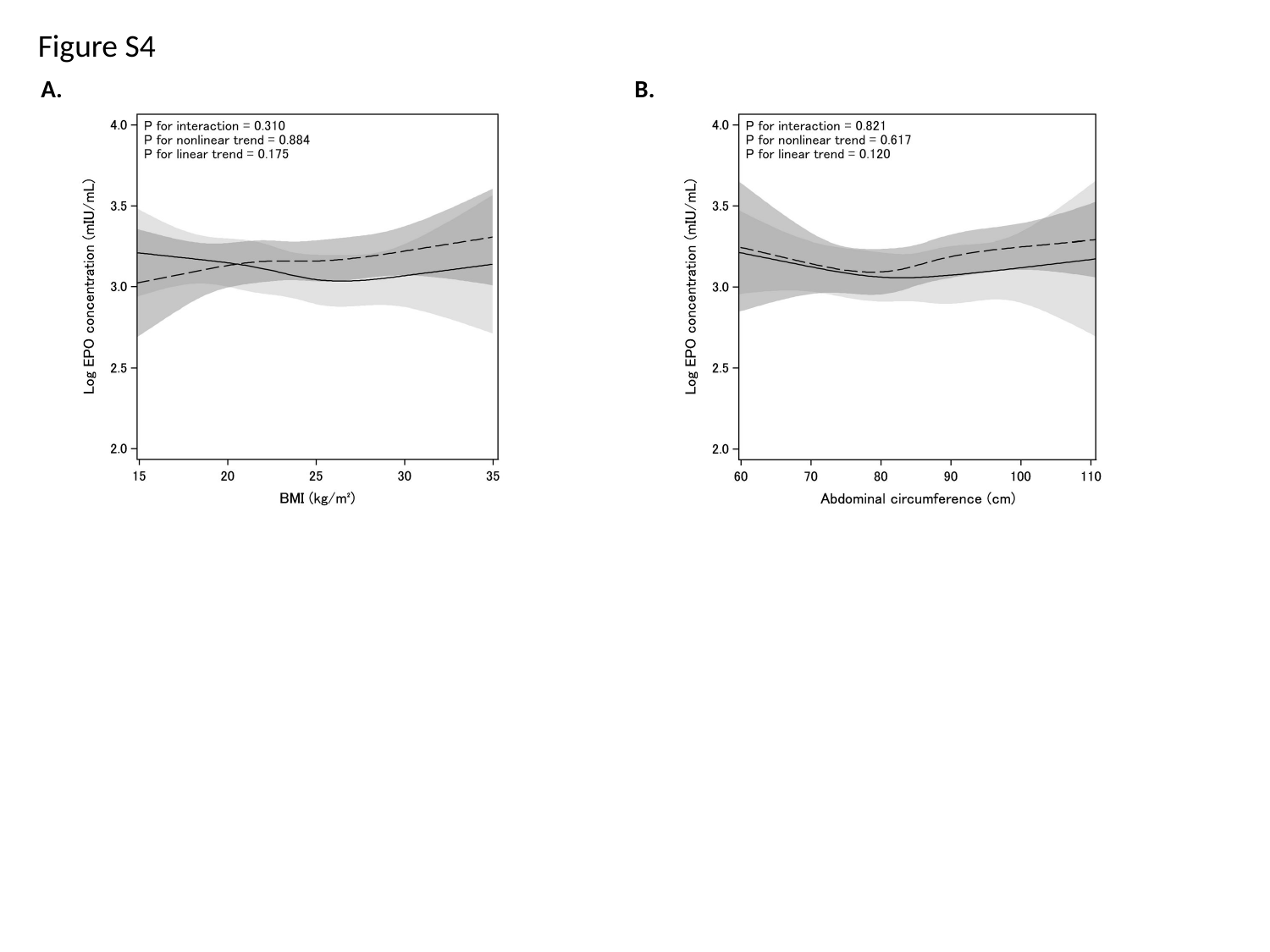

Figure S4
A.
B.

Supplement: Supplementary file 4 — Supplementary Figure S4. Spline curves show the associations between body mass index (BMI) (A) and abdominal circumference (B) and erythropoietin (EPO) level at 1 year, according to sex (solid lines, female patients [BMI, n = 132; abdominal circumference, n = 124]; dashed lines, male patients [BMI, n = 284; abdominal circumference, n = 270]). Spline curves are adjusted for age (in 10-year increments); diabetes mellitus status; chronic kidney disease stage (3, 4, and 5); levels of albumin, log C-reactive protein, log fibroblast growth factor 23, log ferritin, transferrin saturation, albumin-adjusted calcium, and phosphate; medication use (angiotensin-converting enzyme inhibitor inhibitors and angiotensin II receptor blockers); and ferrotherapy use (PPTX 9607 kb) [file 10157_2017_1501_MOESM4_ESM.pptx]

## Slide 1
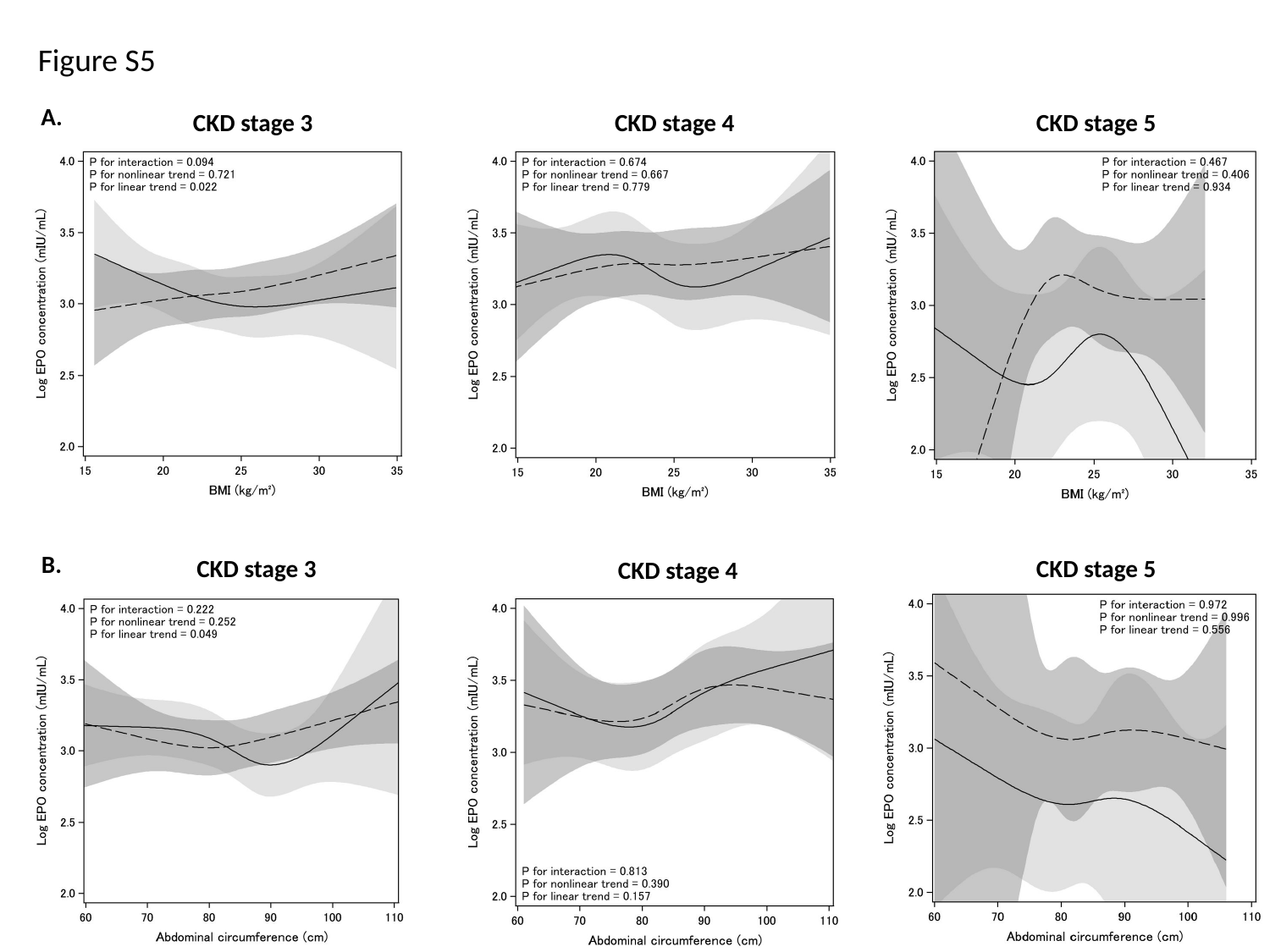

Figure S5
A.
CKD stage 3
CKD stage 4
CKD stage 5
B.
CKD stage 3
CKD stage 5
CKD stage 4

Supplement: Supplementary file 5 — Supplementary Figure S5. Spline curves show the associations between body mass index (BMI) (A) and abdominal circumference (B) and erythropoietin (EPO) levels at 1 year, according to the chronic kidney disease stage (CKD) and sex (solid lines, female patients [BMI: stage 3, n = 63; stage 4, n = 53; stage 5, n = 16], [abdominal circumference: stage 3, n = 62; stage 4, n = 46; stage 5, n = 16]; dashed lines, male patients [BMI: stage 3, n = 143; stage 4, n = 108; stage 5, n = 33], [abdominal circumference: stage 3, n = 133; stage 4, n = 104; stage 5, n = 33]). Spline curves are adjusted for age (in 10-year increments); diabetes mellitus status; levels of albumin, log C-reactive protein, log fibroblast growth factor 23, log ferritin, transferrin saturation, albumin-adjusted calcium, and phosphate; medication use (angiotensin-converting enzyme inhibitor inhibitors and angiotensin II receptor blockers); and ferrotherapy use (PPTX 28742 kb) [file 10157_2017_1501_MOESM5_ESM.pptx]

## Slide 1
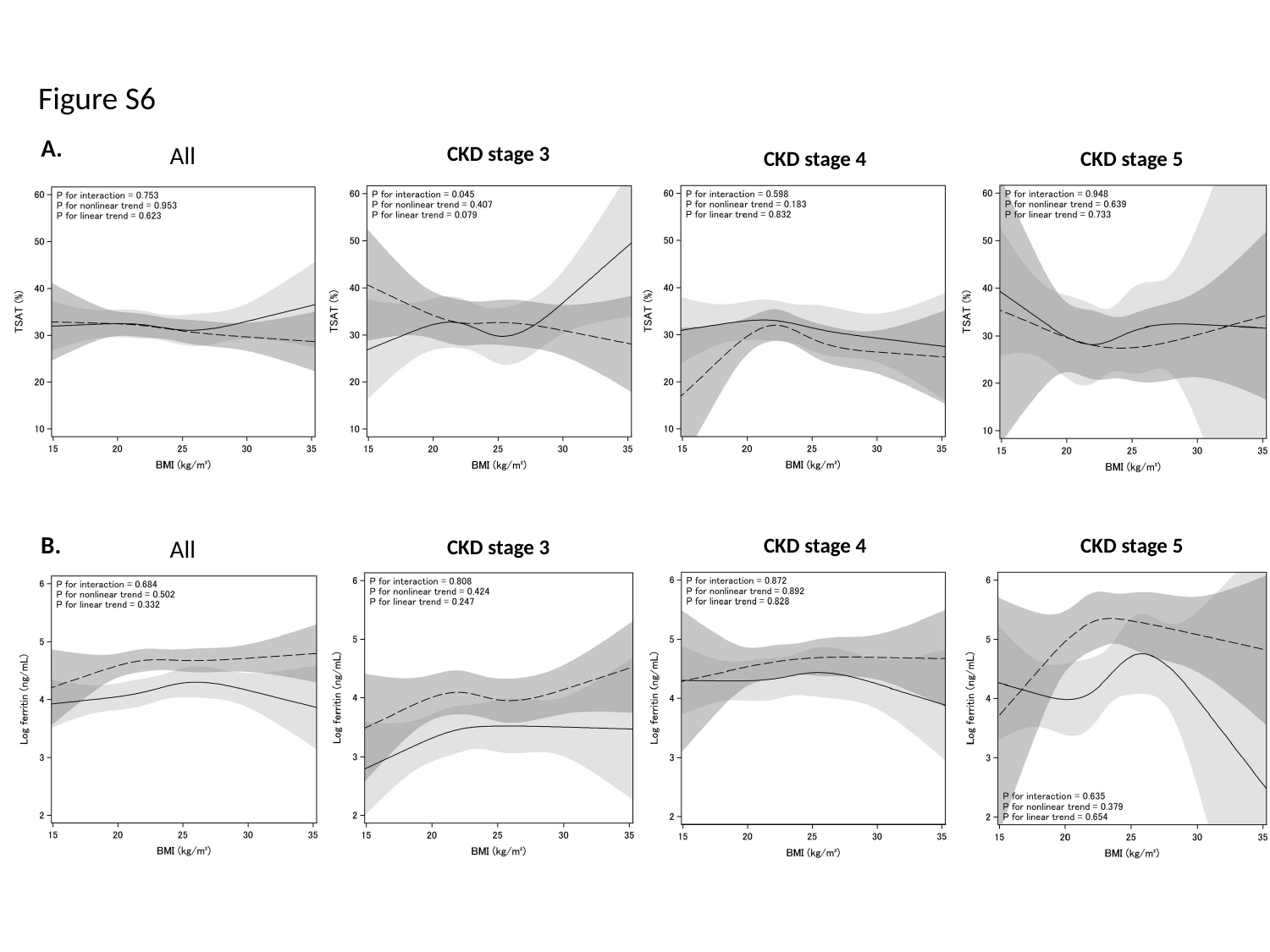

Figure S6
A.
All
CKD stage 3
CKD stage 4
CKD stage 5
B.
CKD stage 4
CKD stage 5
All
CKD stage 3

Supplement: Supplementary file 6 — Supplementary Figure S6. Spline curves show the associations between body mass index (BMI) and transferrin saturation (TSAT) (A) and ferritin levels (B) at baseline, according to sex as well as chronic kidney disease (CKD) stage (solid lines, female patients [TSAT: all, n = 219; stage 3, n = 108; stage 4, n = 82; stage 5, n = 29], [ferritin: all, n = 219; stage 3, n = 108; stage 4, n = 82; stage 5, n = 29]; dashed lines, male patients [TSAT: all, n = 435; stage 3, n = 220; stage 4, n = 162; stage 5, n = 53], [ferritin: all, n = 435; stage 3, n = 220; stage 4, n = 162; stage 5, n = 53]). Spline curves are adjusted for age (in 10-year increments); diabetes mellitus status; CKD stage (3, 4, and 5); levels of albumin, log C-reactive protein, log fibroblast growth factor 23, albumin-adjusted calcium, and phosphate; medication use (angiotensin-converting enzyme inhibitor inhibitors and angiotensin II receptor blockers); and ferrotherapy use. Spline curves are additionally adjusted for log ferritin level in the transferrin saturation analysis, and for transferrin saturation in the ferritin analysis (PPTX 38202 kb) [file 10157_2017_1501_MOESM6_ESM.pptx]

## Slide 1
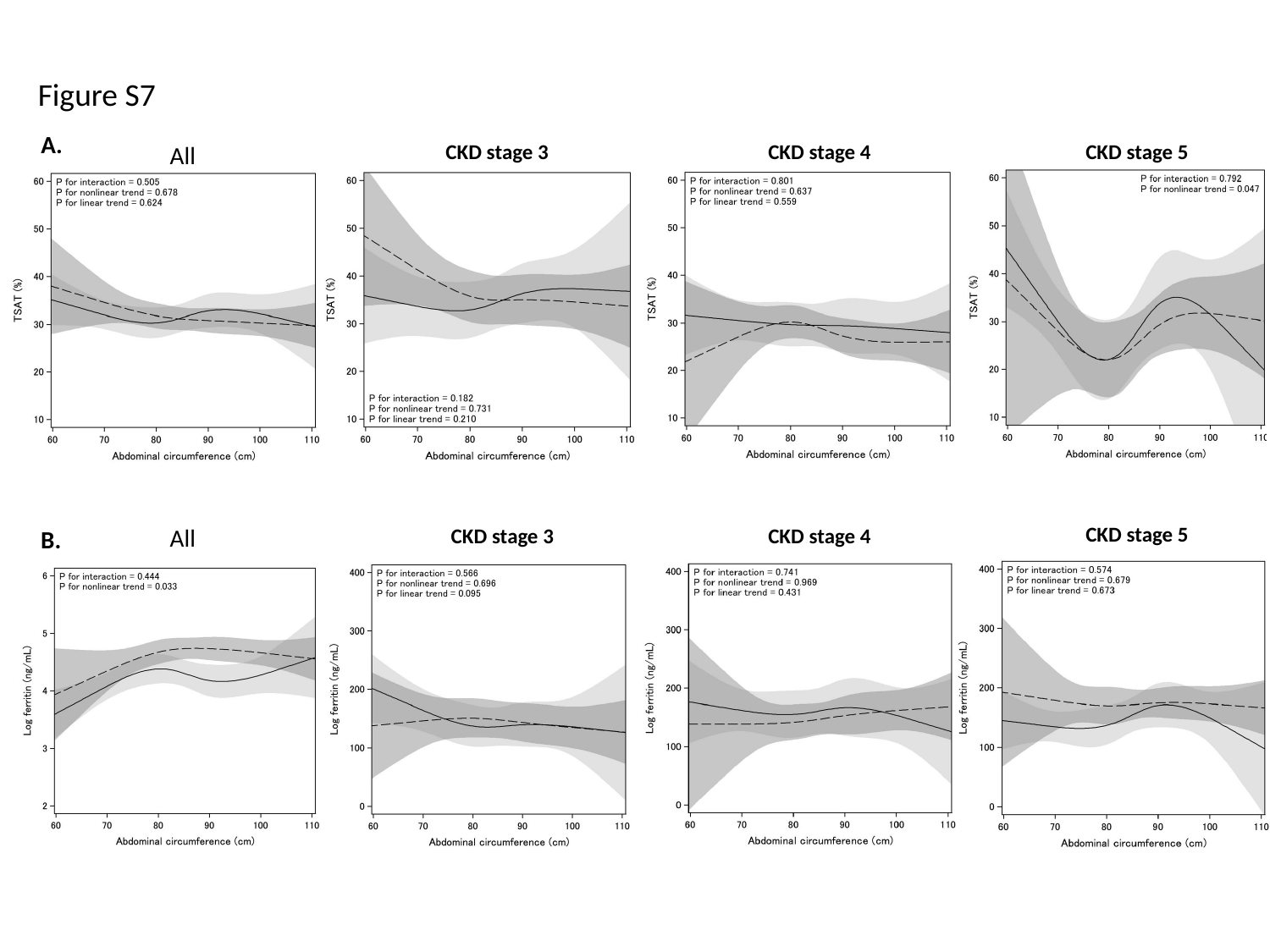

Figure S7
A.
CKD stage 3
CKD stage 4
CKD stage 5
All
CKD stage 5
CKD stage 4
All
CKD stage 3
B.

Supplement: Supplementary file 7 — Supplementary Figure S7. Spline curves show the associations between abdominal circumference and transferrin saturation (TSAT) (A) and ferritin levels (B) at baseline, according to sex as well as chronic kidney disease (CKD) stage (solid lines, female patients [TSAT: all, n = 193; stage 3, n = 98; stage 4, n = 68; stage 5, n = 27], [ferritin: all, n = 193; stage 3, n = 98; stage 4, n = 68; stage 5, n = 27]; dashed lines, male patients [TSAT: all, n = 394; stage 3, n = 194; stage 4, n = 150; stage 5, n = 50], [ferritin: all, n = 394; stage 3, n = 194; stage 4, n = 150; stage 5, n = 50]). Spline curves are adjusted for age (in 10-year increments); diabetes mellitus status; CKD stage (3, 4, and 5); levels of albumin, log C-reactive protein, log fibroblast growth factor 23, albumin-adjusted calcium, and phosphate; medication use (angiotensin-converting enzyme inhibitor inhibitors and angiotensin II receptor blockers); and ferrotherapy use. Spline curves are additionally adjusted for log ferritin level in the transferrin saturation analysis, and for transferrin saturation in the ferritin analysis (PPTX 38051 kb) [file 10157_2017_1501_MOESM7_ESM.pptx]

## Slide 1
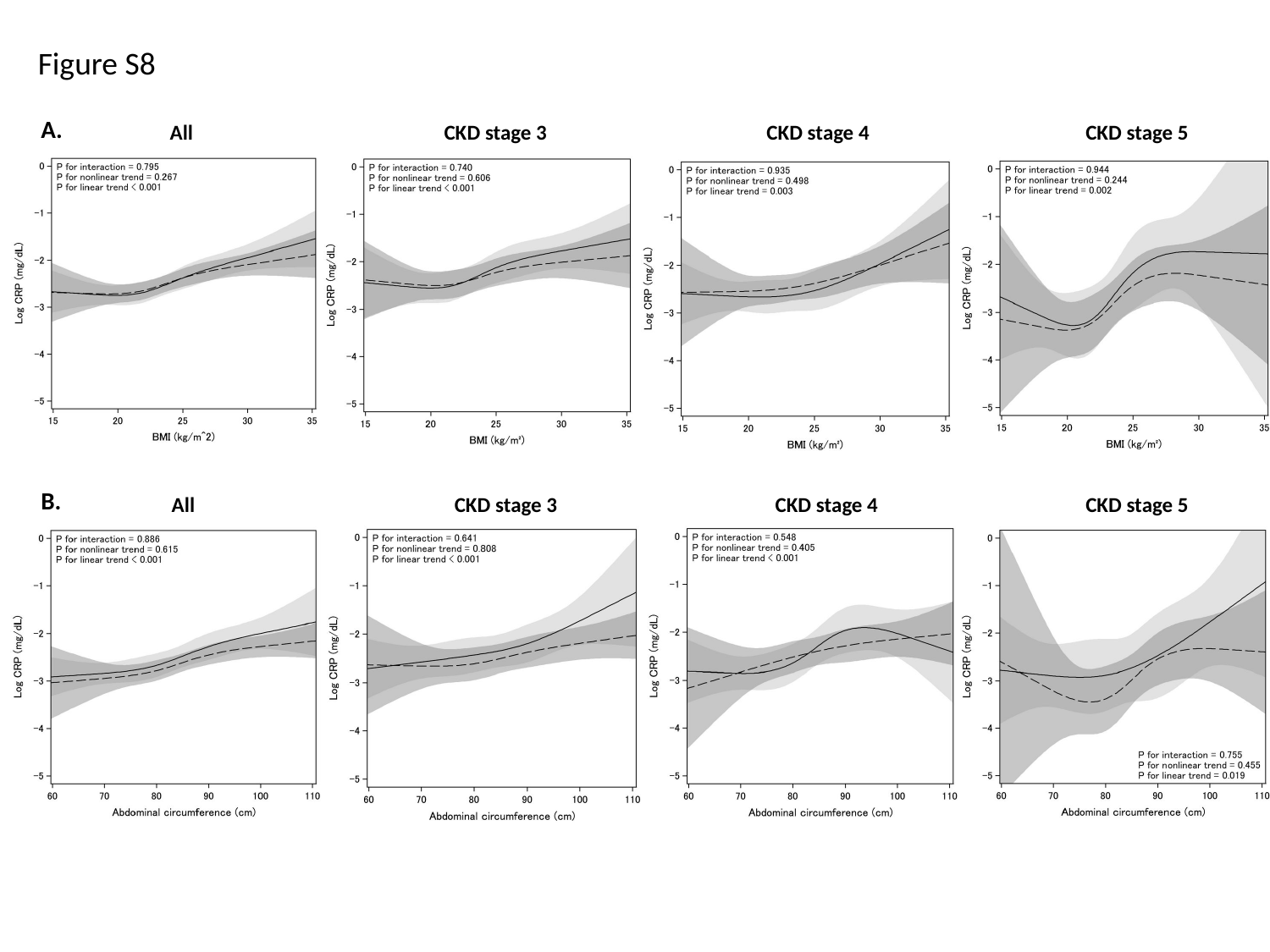

Figure S8
A.
All
CKD stage 3
CKD stage 4
CKD stage 5
B.
All
CKD stage 3
CKD stage 4
CKD stage 5

Supplement: Supplementary file 8 — Supplementary Figure S8. Spline curves show the associations between body mass index (BMI) (A) and abdominal circumference (B) and high-sensitivity C-reactive protein (CRP) level at baseline according to sex as well as chronic kidney disease (CKD) stage (solid lines, female patients [BMI: all, n = 502; stage 3, n = 252; stage 4, n = 186; stage 5, n = 64], [abdominal circumference: all, n = 408; stage 3, n = 205; stage 4, n = 151; stage 5, n = 52]; dashed lines, male patients [BMI: all, n = 963; stage 3, n = 481; stage 4, n = 362; stage 5, n = 120], [abdominal circumference: all, n = 789; stage 3, n = 391; stage 4, n = 299; stage 5, n = 99]). Spline curves are adjusted for age (in 10-year increments); diabetes mellitus status; CKD stage (3, 4, and 5); levels of albumin, log fibroblast growth factor 23, albumin-adjusted calcium, and phosphate; medication use (angiotensin-converting enzyme inhibitor inhibitors and angiotensin II receptor blockers); and ferrotherapy use (PPTX 38197 kb) [file 10157_2017_1501_MOESM8_ESM.pptx]
