# Supplementary material for: Association of adiposity with hemoglobin levels in patients with chronic kidney disease not on dialysis
Source: Clin Exp Nephrol. 2017 Nov 4;22(3):638–46. doi: 10.1007/s10157-017-1501-y (PMC5956024; doi:10.1007/s10157-017-1501-y)
Supplement: Supplementary file 9 — Supplementary material 9 (DOCX 31 kb) [file 10157_2017_1501_MOESM9_ESM.docx]

Table S1-1. Male patient characteristics and laboratory findings according to abdominal circumference

|  | All (n=1069) | Small AC (n=706) | Large AC (n=363) | P value^*^ |
| --- | --- | --- | --- | --- |
| Age (years) | 61.3±10.9 | 61.2±11.0 | 61.4±10.7 | 0.820 |
| Diabetes mellitus (n, %) | 431 (40.3) | 253 (35.8) | 178 (49.0) | <0.001 |
| Height (cm) | 166.3±6.3 [1064] | 165.7±6.2 | 167.5±6.2 | <0.001 |
| Weight (kg) | 61.3±7.6 [1063] | 61.3±7.6 | 76.4±9.8 | <0.001 |
| Body mass index (kg/m^2^) | 23.97±3.44 [1061] | 22.32±2.35 | 27.20±2.89 | <0.001 |
| Abdominal circumference (cm) | 85.2 (80.0–92.0) | 82.0 (78.0–85.0) | 95.0 (91.8–100.0) | <0.001 |
| Cause of CKD (n, %) |  |  |  | 0.005 |
| CGN | 415 (38.8) | 298 (42.2) | 117 (32.2) |  |
| DMN | 236 (22.1) | 139 (19.7) | 97 (26.7) |  |
| Nephrosclerosis | 250 (23.4) | 157 (22.2) | 93 (25.6) |  |
| Other diseases | 168 (15.7) | 112 (15.9) | 56 (15.4) |  |
| CKD stage (n, %) |  |  |  | 0.659 |
| 3A | 135 (12.6) | 88 (12.5) | 47 (12.9) |  |
| 3B | 411 (38.4) | 263 (37.3) | 148 (40.8) |  |
| 4 | 4.5 (37.9) | 275 (39.0) | 130 (35.8) |  |
| 5 | 118 (11.0) | 80 (11.3) | 38 (10.5) |  |
| History of CVD (yes, %) | 271 (25.4) | 164 (23.2) | 107 (29.5) | 0.026 |
| ACE inhibitor / ARB (yes, %) | 906 (84.8) | 584 (82.7) | 322 (88.7) | 0.010 |
| Ferrotherapy (n, %) | 37 (3.5) | 23 (3.3) | 14 (3.9) | 0.612 |
| Red blood cell count (10^4^/μL) | 406.0±60.9 [1050] | 399.3±58.7 | 419.0±63.1 | <0.001 |
| Hemoglobin (g/dL) | 12.76±1.82 [1050] | 12.45±1.74 | 13.18±1.90 | <0.001 |
| Serum albumin (g/dL) | 3.99±0.44 [1042] | 3.98±0.45 | 4.02±0.42 | 0.130 |
| Serum creatinine (mg/dL) | 2.18±1.99 | 2.19±0.99 | 2.17±0.09 | 0.747 |
| eGFR (ml/min/1.73m^2^) | 30.59±12.00 | 30.44±11.94 | 30.87±12.14 | 0.577 |
| Serum cystatin C (mg/L) | 1.797±0.642 [1032] | 1.802±0.632 | 1.789±0.661 | 0.752 |
| Serum corrected calcium (mg/dL) | 9.18±0.42 [967] | 9.20±0.43 | 9.14±0.41 | 0.031 |
| Serum phosphate (mg/dL) | 332±0.63 [949] | 3.30±0.60 | 3.534±0.67 | 0.396 |
| Intact parathyroid hormone (pg/mL) | 73.0 (49.0–111.0) [1032] | 72.0 (49.0–108.0) | 74.0 (49.0–117.0) | 0.404^a^ |
| 25-hydroxyvitamin D (ng/mL) | 16.50 (10.30–24.30) [1011] | 17.40 (10.60–25.85) | 15.10 (9.50–22.40) | 0.002^a^ |
| Fibroblast growth factor 23 (pg/mL) | 55.7 (40.5–85.8) [1018] | 55.3 (39.1–79.7) | 58.0 (42.1–98.9) | 0.031^a^ |
| Serum iron (μg/dL) | 88.5±30.4 [703] | 86.7±29.5 | 92.0±32.0 | 0.030 |
| Total iron binding capacity (μg/dL) | 294.9±50.7 [460] | 290.2±51.4 | 303.8±48.0 | 0.006 |
| Transferrin saturation (%) | 31.45±11.60 [459] | 31.47±11.79 | 31.41±11.26 | 0.954 |
| Serum ferritin (ng/mL) | 116.05 (62.45–192.00) [656] | 115.00 (61.00–188.00) | 116.20 (64.50–201.30) | 0.630^a^ |
| C-reactive protein (mg/dL) | 0.080 (0.040–0.190) [885] | 0.060 (0.030–0.140) | 0.110 (0.050–0.220) | <0.001^a^ |
| Urine albumin-to-creatinine ratio  (mg/gCr) | 502.30 (97.75–1344.90) [1012] | 464.35 (89.45–1267.50) | 562.20 (129.60–1483.85) | 0.044 |

Values are expressed as n (%), mean ± SD or median (interquartile range). *P value for AC-group differences. The number of participants with non-missing data is shown in []; proportions are based on non-missing data. ^a^ P values were calculated using the Kruskal-Wallis test. AC: abdominal circumference, small AC: <90 cm for males and <80 cm for females, large AC: ≥90 cm for males and ≥80 cm for females, CKD: chronic kidney disease, CGN: chronic glomerulonephritis, DMN: diabetic nephropathy, CVD: cardiovascular disease, ACE inhibitor: angiotensin-converting enzyme inhibitor, ARB: angiotensin II receptor blocker, eGFR: estimated glomerular filtration rate
