# Supplementary material for: Association of adiposity with hemoglobin levels in patients with chronic kidney disease not on dialysis
Source: Clin Exp Nephrol. 2017 Nov 4;22(3):638–46. doi: 10.1007/s10157-017-1501-y (PMC5956024; doi:10.1007/s10157-017-1501-y)
Supplement: Supplementary file 10 — Supplementary material 10 (DOCX 32 kb) [file 10157_2017_1501_MOESM10_ESM.docx]

Table S1-2. Female patient characteristics and laboratory findings according to abdominal circumference

|  | All (n=556) | Small AC (n=282) | Large AC (n=274) | P value^*^ |
| --- | --- | --- | --- | --- |
| Age (years) | 58.7±11.8 | 55.9±12.7 | 61.5±10.1 | <0.001 |
| Diabetes mellitus (n, %) | 167 (30.0) | 59 (20.9) | 108 (39.4) | <0.001 |
| Height (cm) | 153.7±6.0 [554] | 154.1±6.1 | 153.2±6.0 | 0.088 |
| Weight (kg) | 54.1±10.3 [555] | 48.0±7.1 | 60.3±9.3 | <0.001 |
| Body mass index (kg/m^2^) | 23.50±3.74 [1867] | 21.65±2.67 | 26.35±3.33 | <0.001 |
| Abdominal circumference (cm) | 79.4 (71.0–88.0) | 71.0 (66.1–75.2) | 88.0 (84.0–95.0) | <0.001 |
| Cause of CKD (n, %) |  |  |  | <0.001 |
| CGN | 319 (57.4) | 168 (59.6) | 151 (55.1) |  |
| DMN | 72 (12.9) | 20 (7.1) | 52 (19.0) |  |
| Nephrosclerosis | 63 (11.3) | 27 (9.6) | 36 (13.1) |  |
| Other diseases | 102 (18.3) | 67 (23.8) | 35 (12.8) |  |
| CKD stage (n, %) |  |  |  | 0.253 |
| 3A | 70 (12.6) | 29 (10.3) | 41 (15.0) |  |
| 3B | 206 (37.1) | 101 (35.8) | 105 (38.3) |  |
| 4 | 216 (38.8) | 117 (41.5) | 99 (36.1) |  |
| 5 | 64 (11.5) | 35 (12.4) | 29 (10.6) |  |
| History of CVD (yes, %) | 77 (13.8) | 31 (11.0) | 46 (16.8) | 0.048 |
| ACE inhibitor / ARB (yes, %) | 434 (78.1) | 203 (72.0) | 231 (84.3) | <0.001 |
| Ferrotherapy (n, %) | 55 (9.9) [556] | 34 (12.1) | 21 (7.7) | 0.083 |
| Red blood cell count (10^4^/μL) | 384.7±55.2 [544] | 377.7±59.2 | 391.9±49.8 | 0.003 |
| Hemoglobin (g/dL) | 11.67±1.43 [544] | 11.46±1.37 | 11.89±1.45 | <0.001 |
| Serum albumin (g/dL) | 4.03±0.38 [543] | 4.05±0.41 | 4.00±0.36 | 0.129 |
| Serum creatinine (mg/dL) | 1.68±0.75 | 1.73±0.76 | 1.62±0.73 | 0.085 |
| eGFR (ml/min/1.73m^2^) | 20.56±12.25 | 30.02±12.27 | 31.12±12.21 | 0.287 |
| Serum cystatin C (mg/L) | 1.710±0.610 [536] | 1.711±0.609 | 1.710±0.612 | 0.991 |
| Serum corrected calcium (mg/dL) | 9.33±0.45 [503] | 9.30±0.45 | 9.35±0.44 | 0.217 |
| Serum phosphate (mg/dL) | 3.74±0.56 [491] | 3.80±0.56 | 3.68±0.56 | 0.020 |
| Intact parathyroid hormone (pg/mL) | 77.0 (54.0–119.0) [537] | 84.0 (54.0–125.0) | 73.0 (55.0–111.0) | 0.184^a^ |
| 25-hydroxyvitamin D (ng/mL) | 13.80 (9.70–18.90) [525] | 14.00 (10.10–19.50) | 13.45 (9.20–18.30) | 0.162^a^ |
| Fibroblast growth factor 23 (pg/mL) | 49.9 (36.8–73.8) [529] | 49.0 (36.5–72.6) | 51.5 (36.9–74.4) | 0.549^a^ |
| Serum iron (μg/dL) | 80.0±31.1 [371] | 80.9±30.4 | 79.1±31.8 | 0.572 |
| Total iron binding capacity (μg/dL) | 302.7±55.5 [244] | 300.8±59.1 | 301.8±51.5 | 0.575 |
| Transferrin saturation (%) | 28.34±11.89 [243] | 28.50±12.47 | 28.17±11.47 | 0.827 |
| Serum ferritin (ng/mL) | 62.00 (35.00–122.30) [345] | 50.80 (27.05–124.35) | 72.00 (41.70–121.00) | 0.034^a^ |
| C-reactive protein (mg/dL) | 0.060 (0.030–0.150) [469] | 0.040 (0.010–0.100) | 0.080 (0.040–0.220) | <0.001^a^ |
| Urine albumin-to-creatinine ratio  (mg/gCr) | 437.70 (114.00–1148.80) [531] | 407.50 (122.60–1046.80) | 482.90 (107.30–1225.40) | 0.344 |

Values are expressed as n (%), mean ± SD or median (interquartile range). *P value for AC-group differences. The number of participants with non-missing data is shown in []; proportions are based on non-missing data. ^a^ P values were calculated using the Kruskal-Wallis test. AC: abdominal circumference, small AC: <90 cm for males and <80 cm for females, large AC: ≥90 cm for males and ≥80 cm for females, CKD: chronic kidney disease, CGN: chronic glomerulonephritis, DMN: diabetic nephropathy, CVD: cardiovascular disease, ACE inhibitor: angiotensin-converting enzyme inhibitor, ARB: angiotensin II receptor blocker, eGFR: estimated glomerular filtration rate
