# Supplementary material for: Association of adiposity with hemoglobin levels in patients with chronic kidney disease not on dialysis
Source: Clin Exp Nephrol. 2017 Nov 4;22(3):638–46. doi: 10.1007/s10157-017-1501-y (PMC5956024; doi:10.1007/s10157-017-1501-y)
Supplement: Supplementary file 11 — Supplementary material 11 (DOCX 27 kb) [file 10157_2017_1501_MOESM11_ESM.docx]

Table S2. Associations between abdominal circumference and hemoglobin level according to sex, as evaluated in multivariate regression models

|  | **Male patients** | | | | | | **Female patients** | | | | |
| --- | --- | --- | --- | --- | --- | --- | --- | --- | --- | --- | --- |
|  | coefficient (95% confidential interval), p value | | | | | | | | | | |
| **Baseline** | Model 1 (n=1050) | | Model 2 (n=392) | Model 3 (n=391) | | | Model 1 (n=544) | | | Model 2 (n=193) | Model 3 (n=189) |
| Small AC | Ref. | | Ref. | Ref. | | | Ref. | | | Ref. | Ref. |
| Large AC | **0.672 (0.468, 0.876)**  **p < 0.001** | | **0.466 (0.165, 0.767)**  **p = 0.003** | **0.475 (0.169, 0.780)**  **p = 0.002** | | | **0.459 (0.225, 0.693)**  **p < 0.001** | | | 0.365 (-0.047, 0.776)  p = 0.082 | 0.331 (-0.099, 0.760)  p = 0.130 |
| **1 year** | Model 1 (n=611) | Model 4 (n=270) | | | | **-** | | Model 1 (n=356) | Model 4 (n=124) | | **-** |
| Small AC | Ref. | | Ref. | | **-** | | Ref. | | | Ref. | Ref. |
| Large AC | **0.687 (0.426, 0.948)**  **p < 0.001** | | **1.014 (0.641, 1.387)**  **p < 0.001** | | **-** | | **0.438 (0.146, 0.730)**  **p = 0.003** | | | 0.200 (-0.316, 0.716)  p = 0.444 | **-** |
| **2 years** | Model 1 (n=449) | Model 2 (n=208) | | | | **-** | | Model 1 (n=280) | Model 2 (n=124) | | **-** |
| Small AC | Ref. | | Ref. | | **-** | | Ref. | | | Ref. | **-** |
| Large AC | **0.743 (0.442, 1.044)**  **p < 0.001** | | **0.840 (0.430, 1.250)**  **p < 0.001** | | - | | **0.512 (0.196, 0.7826)**  **p = 0.002** | | | **0.595 (0.055, 1.134)**  **p = 0.031** | **-** |

Hemoglobin level was the dependent factor in models 1-4. The associations between AC category and hemoglobin level according to sex were adjusted for confounders as follows. Model 1: Age, diabetes mellitus status, and chronic kidney disease stage (3, 4, and 5). Model 2: Albumin level, log C-reactive protein level, transferrin saturation, ferritin level, calcium level corrected by the albumin level, phosphate level, log fibroblast growth factor 23 level, urine albumin-to-creatinine ratio, angiotensin-converting enzyme inhibitor use, angiotensin II receptor blocker use, ferrotherapy use, diet therapy, and the confounders in model 1. Model 3: 25-hydroxyvitamin D level, intact parathyroid hormone level, and the confounders in model 2. Model 4: Albumin level, C-reactive protein level, transferrin saturation, ferritin level, calcium level corrected by the albumin level, phosphate level, fibroblast growth factor 23 level, EPO concentration, angiotensin-converting enzyme inhibitor use, angiotensin II receptor blocker use, erythropoiesis stimulating agent use, ferrotherapy use, diet therapy, and the confounders in models 1. Small AC: <90 cm for males and <80 cm for females, large AC: ≥90 cm for males and ≥80 cm for females
