# Supplementary material for: Association of adiposity with hemoglobin levels in patients with chronic kidney disease not on dialysis
Source: Clin Exp Nephrol. 2017 Nov 4;22(3):638–46. doi: 10.1007/s10157-017-1501-y (PMC5956024; doi:10.1007/s10157-017-1501-y)
Supplement: Supplementary file 13 — Supplementary material 13 (DOCX 29 kb) [file 10157_2017_1501_MOESM13_ESM.docx]

Table S3-2. Characteristics and laboratory data of patients included and excluded from Model 2, but included in Model 1, in the multivariate regression analysis of the association between abdominal circumference and hemoglobin level

|  | Male patients | | Female patients | | |
| --- | --- | --- | --- | --- | --- |
|  | Excluded (N=658) | Included (N=392) | Excluded (N=351) | | Included (N=193) |
| Age (years) | 61.2±11.0 | 61.6±10.5 | 58.2±11.4 | 59.6±12.5 | |
| Diabetes mellitus (n, %) | 275 (41.8) | 152 (38.8) | 110 (31.3) | 54 (28.0) | |
| Height (cm) | 166.3±6.2 | 166.1±6.4 | 154.0±6.2 | 153.0±5.7 | |
| Weight (kg) | 66.2±10.9 | 66.7±11.3 | 54.3±10.4 | 53.7±10.2 | |
| Body mass index (kg/m^2^) | 23.52 (21.57–25.82) | 23.76 (21.84–26.06) | 22.41 (20.13–25.11) | 22.81 (19.56–25.91) | |
| Abdominal circumference (cm) | 86.2±9.7 | 87.2±9.5 | 79.3±11.8 | 81.3±11.8 | |
| Cause of CKD (n, %) |  |  |  |  | |
| CGN | 153 (23.3) | 82 (20.9) | 52 (14.8) | 18 (9.3) | |
| DMN | 243 (36.9) | 163 (41.6) | 199 (56.7) | 115 (59.6) | |
| Nephrosclerosis | 149 (22.6) | 97 (24.7) | 33 (9.4) | 27 (14.0) | |
| Other diseases | 113 (17.2) | 50 (12.8) | 67 (19.1) | 33 (17.1) | |
| CKD stage (n, %) |  |  |  |  | |
| 3A | 89 (13.5) | 43 (11.0) | 35 (10.0) | 33 (17.1)* | |
| 3B | 251 (38.1) | 150 (38.3) | 134 (38.2) | 65 (33.7) | |
| 4 | 250 (38.0) | 149 (38.0) | 146 (41.6) | 68 (35.2) | |
| 5 | 68 (10.3) | 50 (12.8) | 36 (10.3) | 27 (14.0) | |
| History of CVD (yes, %) | 165 (25.1) | 106 (27.0) | 48 (13.7) | 27 (14.0) | |
| ACE inhibitor / ARB (yes, %) | 548 (83.3) | 341 (87.0) | 278 (79.2) | 148 (76.7) | |
| Ferrotherapy (n, %) | 17 (2.6) | 20 (5.1)* | 34 (9.7) | 21 (10.9) | |
| Red blood cell count (10^4^/μL) | 404.7±61.1 | 408.1±60.6 | 383.0±48.9 | 387.7±65.0 | |
| Hemoglobin (g/dL) | 12.70±1.82 | 12.86±1.81 | 11.59±1.42 | 11.81±1.43 | |
| Serum albumin (g/dL) | 3.97±0.46 | 4.04±0.40* | 4.01±0.36 | 4.05±0.43 | |
| Serum creatinine (mg/dL) | 2.16±0.96 | 2.24±1.04 | 1.69±0.72 | 1.68±0.80 | |
| eGFR (ml/min/1.73m^2^) | 30.91±12.16 | 29.87±11.75 | 30.01±11.52 | 31.18±13.41 | |
| Serum cystatin C (mg/L) | 1.787±0.631 | 1.826±0.661 | 1.719±0.593 | 1.719±0.639 | |
| Serum corrected calcium (mg/dL) | 9.17±0.43 | 9.17±0.40 | 9.31±0.44 | 9.34±0.46 | |
| Serum phosphate (mg/dL) | 3.31±0.57 | 3.33±0.70 | 3.75±0.54 | 3.73±0.59 | |
| Intact parathyroid hormone (pg/mL) | 73.0 (49.0–116.0) | 73.0 (49.0–107.0) | 76.0 (55.0–113.5) | 81.0 (53.0–134.0) | |
| 25-hydroxyvitamin D (ng/mL) | 16.25 (10.20–24.10) | 17.20 (10.80–24.80) | 13.75 (9.75–17.95) | 13.70 (9.30–20.90) | |
| Fibroblast growth factor 23 (pg/mL) | 55.9 (39.8–86.8) | 55.9 (40.9–86.2) | 50.1 (36.9–73.5) | 52.2 (37.4–76.4) | |
| Serum iron (μg/dL) | 85.0±28.9 | 91.4±31.4* | 76.2±28.4 | 83.5±33.1* | |
| Total iron binding capacity (μg/dL) | 286.6±44.0 | 296.3±51.7 | 301.7±41.3 | 302.7±58.8 | |
| Transferrin saturation (%) | 31.50±12.47 | 31.45±11.47 | 27.37±11.13 | 28.67±12.21 | |
| Serum ferritin (ng/mL) | 115.50 (58.25–182.50) | 115.90 (64.55–197.50) | 55.50 (35.20–114.00) | 71.30 (32.70–126.20) | |
| C-reactive protein (mg/dL) | 0.090 (0.040–0.200) | 0.070 (0.040–0.150)* | 0.060 (0.030–0.170) | 0.060 (0.030–0.120) | |
| Urine albumin-to-creatinine ratio  (mg/gCr) | 547.80 (121.90–1353.00) | 459.95 (76.95–1312.85) | 430.20 (122.40–1158.40) | 437.80 (109.10–1115.40) | |

Values are expressed as n (%), mean ± SD or median (interquartile range). *: The difference between excluded and included patients (within sex) is statistically significant. Proportions are based on non-missing data. CKD: chronic kidney disease, CGN: chronic glomerulonephritis, DMN: diabetic nephropathy, CVD: cardiovascular disease, ACE inhibitor: angiotensin-converting enzyme inhibitor, ARB: angiotensin II receptor blocker, eGFR: estimated glomerular filtration rate
