# Supplementary material for: Association of adiposity with hemoglobin levels in patients with chronic kidney disease not on dialysis
Source: Clin Exp Nephrol. 2017 Nov 4;22(3):638–46. doi: 10.1007/s10157-017-1501-y (PMC5956024; doi:10.1007/s10157-017-1501-y)
Supplement: Supplementary file 14 — Supplementary material 14 (DOCX 27 kb) [file 10157_2017_1501_MOESM14_ESM.docx]

Table S4. Associations between body mass index and hemoglobin level at baseline and across time, according to sex

|  | **Male patients** | | | **Female patients** | | |
| --- | --- | --- | --- | --- | --- | --- |
| **Cross-sectional**  **effect (baseline)** | Model 1 (n=1497) | Model 2 (n=432) | Model 3 (n=431) | Model 1 (n=825) | Model 2 (n=220) | Model 3 (n=215) |
|  | Coefficient, 95% confidential interval, p value | | | | | |
| Low BMI | -0.332 (-0.763, 0.099)  p = 0.131 | **-0.976 (-1.789, -0.163)**  **p = 0.019** | **1.001 (-1.865~, -0.138)**  **p = 0.023** | **-0.344 (-0.639, 0.050)**  **p = 0.022** | **-0.589 (-1.145, -0.034)**  **p = 0.038** | -0.561 (-1.131, 0.009)  p = 0.054 |
| Normal BMI | Ref | Ref | Ref | Ref | Ref | Ref |
| High BMI | **0.690 (0.521, 0.859)**  **p < 0.001** | **0.578 (0.298, 0.858)**  **p<0.001** | **0.584 (0.302, 0.867)**  **p<0.001** | **0.337 (0.122, 0.552)**  **p = 0.002** | 0.039 (-0.381, 0.459)  p = 0.855 | 0.026 (-0.404, 0.456)  p = 0.905 |
| **Longitudinal effect** | Model 1 | Model 2 | Model 3 | Model 1 | Model 2 | Model 3 |
|  | Coefficient, 95% confidential interval, p value | | | | | |
| Low BMI | -0.086 (-0.303, 0.132)  p = 0.440 | 0.025 (-0.441, 0.492)  p = 0.915 | 0.074 (-0.432, 0.580)  p = 0.773 | -0.119 (-0.269, 0.031)  p = 0.120 | -0.103 (-0.394, 0.189)  p = 0.488 | 0.016 (-0.262, 0.294)  p = 0.907 |
| Normal BMI | Ref | Ref | Ref | Ref | Ref | Ref |
| High BMI | **-0.118 (-0.201, 0.034)**  **p = 0.006** | -0.009 (-0.165, 0.147)  p = 0.909 | 0.001 (-0.155, 0.158)  p = 0.989 | -0.073 (-0.179, 0.033)  p = 0.178 | 0.108 (-0.114, 0.330)  p = 0.338 | 0.136 (-0.077, 0.349)  p = 0.209 |

Hemoglobin level was the dependent factor, and baseline covariates were used in models 1-3. The associations between BMI category and hemoglobin level according to sex were adjusted for confounders as follows. Model 1: Age, diabetes mellitus, and chronic kidney disease stage (3, 4, and 5). Model 2: Albumin level, log C-reactive protein level, transferrin saturation, ferritin level, calcium level corrected by the albumin level, phosphate level, log fibroblast growth factor 23 level, urine albumin-to-creatinine ratio, angiotensin-converting enzyme inhibitor use, angiotensin II receptor blocker use, ferrotherapy use, diet therapy, and the confounders in models 1. Model 3: 25-hydroxyvitamin D level, intact parathyroid hormone level, and the confounders in model 2. Low BMI: <18.5 kg/m^2^, normal BMI: 18.5-24.5 kg/m^2^, high BMI: ≥25 kg/m^2^
