# Supplementary material for: Association of adiposity with hemoglobin levels in patients with chronic kidney disease not on dialysis
Source: Clin Exp Nephrol. 2017 Nov 4;22(3):638–46. doi: 10.1007/s10157-017-1501-y (PMC5956024; doi:10.1007/s10157-017-1501-y)
Supplement: Supplementary file 15 — Supplementary material 15 (DOCX 24 kb) [file 10157_2017_1501_MOESM15_ESM.docx]

Table S5. Associations between abdominal circumference and hemoglobin level at baseline and across time, according to sex

|  | **Male patients** | | | | **Female patients** | | |  |
| --- | --- | --- | --- | --- | --- | --- | --- | --- |
| **Cross-sectional effect**  **(baseline)** | Model 1 (n=1069) | Model 2 (n=392) | Model 3 (n=391) | Model 1 (n=556) | | Model 2 (n=194) | Model 3 (n=190) |  |
|  | Coefficient, 95% confidential interval, p value | | | | | | |  |
| Small AC | Ref | Ref | Ref | Ref | | Ref | Ref |  |
| Large AC | **0.660 (0.459, 0.860)**  **p < 0.001** | **0.479 (0.181, 0.777)**  **p = 0.002** | **0.482 (0.179, 0.785)**  **p = 0.002** | **0.430 (0.200, 0.661)**  **p < 0.001** | | 0.386 (-0.021, 0.794)  p = 0.063 | 0.365 (-0.061, 0.790)  p = 0.093 |  |
| **Longitudinal effect** | Model 1 | Model 2 | Model 3 | Model 1 | | Model 2 | Model 3 |  |
|  | Coefficient, 95% confidential interval, p value) | | | | | | |  |
| Small AC | Ref | Ref | Ref | Ref | | Ref | Ref |  |
| Large AC | -0.019 (-0.116, 0.078)  p = 0.705 | 0.075 (-0.093, 0.244)  p = 0.380 | 0.081 (-0.090, 0.252)  p = 0.353 | 0.024 (-0.087, 0.136)  p = 0.667 | | 0.100 (-0.123, 0.323)  p = 0.378 | 0.085 (-0.131, 0.301)  p = 0.439 |  |

Hemoglobin level was the dependent factor, and baseline covariates were used in models 1-3. The associations between AC category and hemoglobin level according to sex were adjusted for con-founders as follows. Model 1: Age, diabetes mellitus status, and chronic kidney disease stage (3, 4, and 5). Model 2: Albumin level, log C-reactive protein level, transferrin saturation, ferritin level, calcium level corrected by the albumin level, phosphate level, log fibroblast growth factor 23 level, angiotensin-converting enzyme inhibitor use, angiotensin II receptor blocker use, ferrotherapy use, diet therapy, and the confounders in model 1. Model 3: 25-hydroxyvitamin D level, intact parathyroid hormone level, and the confounders in model 2. Small AC: <90 cm for males and <80 cm for females, large AC: ≥90 cm for males and ≥80 cm for females
