# Supplementary material for: Association of adiposity with hemoglobin levels in patients with chronic kidney disease not on dialysis
Source: Clin Exp Nephrol. 2017 Nov 4;22(3):638–46. doi: 10.1007/s10157-017-1501-y (PMC5956024; doi:10.1007/s10157-017-1501-y)
Supplement: Supplementary file 16 — Supplementary material 16 (DOCX 22 kb) [file 10157_2017_1501_MOESM16_ESM.docx]

Table S6. Erythropoietin concentration at 1 year of follow-up, according to body composition categories and sex

| Body mass index category | | | | | | | | |  | | |
| --- | --- | --- | --- | --- | --- | --- | --- | --- | --- | --- | --- |
|  | Female | | All N=537 | | Low BMI (N=60) | Normal BMI (N=331) | | High BMI (N=146) | | | P value |
|  | EPO concentration (mIU/mL) | | 21.50 (17.35, 26.80) [484] | | 24.55 (19.70, 31.50) | 20.70 (16.90, 25.90) | | 21.70 (18.50, 26.70) | | | 0.002^a^ |
|  | Male | | All N=965 | | Low BMI (N=39) | Normal BMI (N=599) | | High BMI (N=327) | | | P value |
|  | EPO concentration (mIU/mL) | | 21.40 (17.70, 27.00) [873] | | 22.55 (18.00, 27.55) | 21.05 (17.60, 26.80) | | 21.80 (17.90, 27.20) | | | 0.507^a^ |
| Abdominal circumference category | | | | | | | | |  | | |
|  | Female | All N=368 | | Small AC (N=160) | | | Large AC (N=208) | | | P value | |
|  | EPO concentration (mIU/mL) | 20.90 (17.30, 26.50) [344] | | 20.60 (17.10, 25.90) | | | 21.00 (17.50, 26.70) | | | 0.562^a^ | |
|  | Male | All N=631 | | Small AC (N=421) | | | Large AC (N=210) | | | P value | |
|  | EPO concentration (mIU/mL) | 21.55 (17.70, 27.10) [602] | | 21.25 (17.40, 26.50) | | | 22.60 (18.70, 28.00) | | | 0.018^a^ | |

Values are expressed as median (interquartile range). The number of participants with non-missing data is shown in []. ^a^: P value was calculated using the Kruskal-Wallis test. EPO, erythropoietin, BMI: body mass index, low BMI: <18.5 kg/m^2^, normal BMI: 18.5-24.5 kg/m^2^, high BMI: ≥25 kg/m^2^, AC: abdominal circumference, small AC: <90 cm for males and <80 cm for females, large AC: ≥90 cm for males and ≥80 cm for females
