# Supplementary material for: Association of adiposity with hemoglobin levels in patients with chronic kidney disease not on dialysis
Source: Clin Exp Nephrol. 2017 Nov 4;22(3):638–46. doi: 10.1007/s10157-017-1501-y (PMC5956024; doi:10.1007/s10157-017-1501-y)
Supplement: Supplementary file 17 — Supplementary material 17 (DOCX 25 kb) [file 10157_2017_1501_MOESM17_ESM.docx]

Table S7. Associations between body composition category and erythropoietin level at 1 year of follow-up, according to sex

|  | | **Male patients** | | **Female patients** | | |
| --- | --- | --- | --- | --- | --- | --- |
|  | **Erythropoietin** (Coefficient, 95% confidential interval, p value | | | | | |
|  | Model 1 (n=1087) | | Model 2 (n=288) | | Model 1 (n=621) | Model 2 (n=136) |
| Low BMI | 0.024 (-0.094, 0.143)  p = 0.688 | | -0.058 (-0.279, 0.163)  p = 0.604 | | **0.157 (0.050, 0.264)**  **p = 0.004** | 0.180 (-0.028, 0.389)  p = 0.090 |
| Normal BMI | Ref. | | Ref. | | Ref. | Ref. |
| High BMI | 0.039 (-0.015, 0.094)  p = 0.158 | | -0.009 (-0.110, 0.092)  p = 0.859 | | **0.091 (0.010, 0.173)**  **p = 0.027** | 0.028 (-0.121, 0.179)  p = 0.707 |
|  | Model 3 (n=829) | | Model 4 (n=280) | | Model 3 (n=439) | Model 4 (n=132) |
| Small AC | Ref. | | Ref. | | Ref. | Ref. |
| Large AC | **0.098 (0.043, 0.154)**  **p < 0.001** | | 0.094 (-0.005, 0.193)  p = 0.064 | | **0.096 (0.040, 0.151)**  **p < 0.001** | -0.002 (-0.158, 0.154)  p = 0.983 |

Serum erythropoietin level was the dependent factor in models 1-4. The associations between BMI/AC category and erythropoietin level according to sex were adjusted for confounders as follows. Models 1 and 3: Age, diabetes mellitus status, and chronic kidney disease stage (3, 4, and 5). Models 2 and 4: Albumin level, log C-reactive protein level, transferrin saturation, ferritin level, calcium level corrected by the albumin level, phosphate level, log fibroblast growth factor 23 level, angiotensin-converting enzyme inhibitor use, angiotensin II receptor blocker use, ferrotherapy use, diet therapy, and the confounders in models 1 and 3.
