# Supplementary material for: Association of adiposity with hemoglobin levels in patients with chronic kidney disease not on dialysis
Source: Clin Exp Nephrol. 2017 Nov 4;22(3):638–46. doi: 10.1007/s10157-017-1501-y (PMC5956024; doi:10.1007/s10157-017-1501-y)
Supplement: Supplementary file 19 — Supplementary material 19 (DOCX 30 kb) [file 10157_2017_1501_MOESM19_ESM.docx]

Table S9. Associations between abdominal circumference and transferrin saturation and ferritin levels according to sex, as evaluated in multivariate regression models

|  | **Male patients** | | | | **Female patients** | | | |
| --- | --- | --- | --- | --- | --- | --- | --- | --- |
|  | **Transferrin saturation** (coefficient, 95% confidential interval, p value) | | | | | | |  |
| **Baseline** | Model 1 (n=459) | Model 2 (n=394) | | Model 3 (n=393) | Model 1 (n=243) | Model 2 (n=193) | Model 3 (n=189) |  |
| Small AC | Ref. | Ref. | | Ref. | Ref. | Ref. | Ref. |  |
| Large AC | -0.015 (-0.244, 0.214)  p = 0.897 | -0.107 (-0.341, 0.127)  p = 0.371 | | -0.086 (-0.321, 0.150)  p = 0.475 | -0.027 (-0.349, 0.295)  p = 0.870 | 0.074 (-0.298, 0.445)  p = 0.696 | 0.058 (-0.326, 0.441)  p = 0.767 |  |
| **1 year** | Model 1 (n=295) | Model 2 (n=270) | | **-** | Model 1 (n=149) | Model 2 (n=124) | **-** |  |
| Small AC | Ref. | Ref. | | **-** | Ref. | Ref. | **-** |  |
| Large AC | 0.006 (-0.286, 0.299)  p = 0.965 | 0.078 (-0.222, 0.378)  p = 0.610 | | **-** | 0.113 (-0.280, 0.506)  p = 0.571 | 0.028 (-0.411, 0.466)  p = 0.901 | **-** |  |
| **2 years** | Model 1 (n=233) | Model 2 (n=210) | | **-** | Model 1 (n=131) | Model 2 (n=115) | **-** |  |
| Small AC | Ref. | Ref. | | **-** | Ref. | Ref. | **-** |  |
| Large AC | 0.104 (-0.210, 0.419)  p = 0.514 | -0.019 (-0.362, 0.324)  p = 0.913 | | **-** | -0.012 (-0.420, 0.395)  p = 0.953 | 0.206 (-0.230, 0.642)  p = 0.352 | **-** |  |
|  | **Ferritin** (coefficient, 95% confidential interval, p value) | | | | | | |  |
| **Baseline** | Model 4 (n=656) | Model 5 (n=394) | | Model 6 (n=393) | Model 4 (n=345) | Model 5 (n=193) | Model 6 (n=189) |  |
| Small AC | Ref. | Ref. | | Ref. | Ref. | Ref. | Ref. |  |
| Large AC | 0.016 (-0.126, 0.158)  p = 0.824 | 0.050 (-0.135, 0.235)  p = 0.597 | | 0.065 (-0.123, 0.253)  p = 0.496 | 0.026 (-0.182, 0.233)  p = 0.808 | -0.164 (-0.440, 0.113)  p = 0.244 | -0.180 (-0.469, 0.109)  p = 0.220 |  |
| **1 year** | Model 4 (n=389) | Model 5 (n=270) | | - | Model 4 (n=214) | Model 5 (n=124) |  |  |
| Small AC | Ref. | Ref. | | **-** | Ref. | Ref. | **-** |  |
| Large AC | -0.048 (-0.237, 0.142)  p = 0.622 | -0.193 (-0.421, 0.035)  p = 0.097 | | **-** | 0.232 (-0.022, 0.485)  p = 0.073 | 0.019 (-0.335, 0.372)  p = 0.917 | **-** |  |
| **2 years** | Model 4 (n =306) | | Model 5 (n = 210) |  | Model 4 (n =185) | Model 5 (n = 115) |  |  |
| Small AC | Ref. | | Ref. | **-** | Ref. | Ref. | **-** |  |
| Large AC | 0.169 (-0.029, 0.367)  p = 0.095 | | 0.135 (-0.111, 0.380)  p = 0.280 | **-** | 0.121 (-0.137, 0.379)  p = 0.356 | 0.144 (-0.234, 0.522)  p = 0.452 | **-** |  |

Transferrin saturation was the dependent factor in models 1-3, and ferritin level was the dependent factor in models 4-6. The associations between AC category and transferrin saturation/ferritin level according to sex were adjusted for confounders as follows. Models 1 and 4: Age, diabetes mellitus status, and chronic kidney disease stage (3, 4, and 5). Model 2: Albumin level, log C-reactive protein level, ferritin level, calcium level corrected by the albumin level, phosphate level, log fibroblast growth factor 23 level, angiotensin-converting enzyme (ACE) inhibitor use, angiotensin II receptor blocker (ARB) use, ferrotherapy use, diet therapy, and the confounders in model 1. Model 3: 25-hydroxyvitamin D level, intact parathyroid hormone level, and the confounders in model 2. Model 5: Albumin level, log C-reactive protein level, transferrin saturation, calcium level corrected by the albumin level, phosphate level, log fibroblast growth factor 23 level, ACE inhibitor use, ARB use, ferrotherapy use, diet therapy, and the confounders in model 4. Model 6: 25-hydroxyvitamin D level, intact parathyroid hormone level, and the confounders in model 5.
